# Process Technologies for Disinfection of Food-Contact Surfaces in the Dry Food Industry: A Review

**DOI:** 10.3390/microorganisms13030648

**Published:** 2025-03-12

**Authors:** Harleen Kaur Dhaliwal, Shivani Sonkar, Prithviraj V, Luis Puente, M. S. Roopesh

**Affiliations:** 1 Department of Agricultural, Food and Nutritional Science, University of Alberta, Edmonton, AB T6G 2P5, Canada; harleen.dhaliwal@ualberta.ca (H.K.D.); prithvi1@ualberta.ca (P.V.);; 2 Departamento de Ciencias de los Alimentos y Tecnología Química, Facultad de Ciencias Químicas y Farmacéuticas, Universidad de Chile, Av. Dr. Carlos Lorca Tobar 964, Independencia, Santiago 8380494, Chile

**Keywords:** dry food safety, food-contact surfaces, microbial contamination, disinfection strategies, water activity

## Abstract

The survival characteristics of bacterial pathogens, including *Salmonella* spp., *Listeria monocytogenes*, *Staphylococcus aureus,* and *Escherichia coli,* in foods with a low water activity (*a_w_*) have been extensively examined and reported. Microbial attachment on the food-contact surfaces can result in cross-contamination and compromise the safety of low-*a_w_* foods. The bactericidal potential of various conventional and novel disinfection technologies has been explored in the dry food industry. However, the attachment behavior of bacterial pathogens to food-contact surfaces in low-*a_w_* conditions and their subsequent response to the cleaning and disinfection practices requires further elucidation. The review summarizes the elements that influence disinfection, such as the presence of organic residues, persistent strains, and the possibility of microbial biotransfer. This review explores in detail the selected dry disinfection technologies, including superheated steam, fumigation, alcohol-based disinfectants, UV radiation, and cold plasma, that can be used in the dry food industry. The review also highlights the use of several wet disinfection technologies employing chemical antimicrobial agents against surface-dried microorganisms on food-contact surfaces. In addition, the disinfection efficacy of conventional and novel technologies against surface-dried microorganisms on food-contact surfaces, as well as their advantages and disadvantages and underlying mechanisms, are discussed. Dry food processing facilities should implement stringent disinfection procedures to ensure food safety. Environmental monitoring procedures and management techniques are essential to prevent adhesion and allow the subsequent inactivation of microorganisms.

## 1. Introduction

Low-water-activity (*a_w_*) foods are those with an *a_w_* of less than 0.85 [1]. Previous studies have reported the presence and survival of pathogenic microorganisms in low-*a_w_* foods [2,3]. Recent low-*a_w_* food product recalls due to the presence of various pathogenic microorganisms in flour [4], peanut butter [5], tahini [6], dried coconut [7], and chia seeds [8] have generated significant alarm. Microorganisms, including *Salmonella* spp., *L. monocytogenes*, *E. coli*, and *S. aureus*, can survive under low-*a_w_* conditions [9,10,11]. Rehydration of the contaminated low-*a_w_* foods creates a favorable environment for microorganisms, thereby increasing the likelihood of infection. The desiccation of microorganisms in a low*-a_w_* environment in the dry food processing industry renders them resistant to various disinfection processes and causes food safety issues [3]. Cross-contamination and poor sanitation are key contributors to foodborne outbreaks [12]. Surface-adhered microorganisms may detach and colonize nearby surfaces and cross-contaminate foods [13,14]. Furthermore, the existence of persistent strains in food processing has a significant effect on subsequent contamination [15]. Table 1 outlines the outbreak investigations linked to the contaminated dry food production environments.

**Figure 1:**
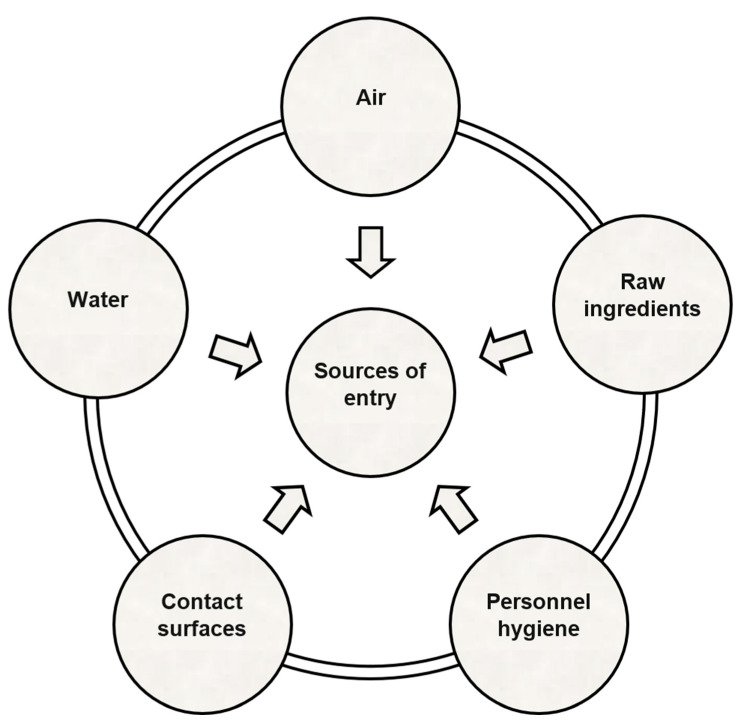
Possible contamination routes of microorganisms into the dry food processing facility.

**Figure 2:**
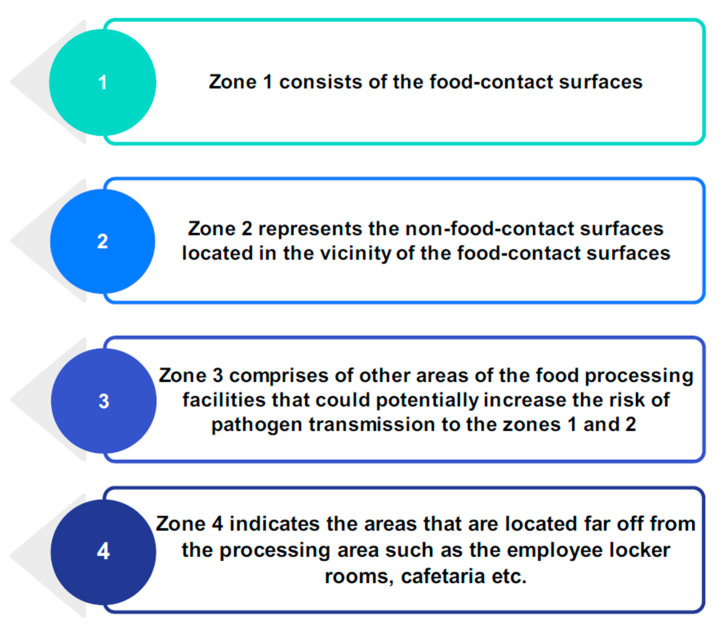
Hygienic zoning in food processing facility (modified from [35]).

Microorganisms can enter food manufacturing facilities and industrial environments via airflow, water, personnel, or raw materials (Figure 1) [32]. Open and closed-flow systems make up a food processing plant. The open systems of the food processing plants comprise cutting boards, knives, slicers, and conveyor belts. The food utilized in such systems may be solid or liquid and is exposed to air. The system creates favorable conditions for bacterial adhesion and growth. Closed-flow systems are those used for carrying liquid and solid materials, such as pipelines, vessels, reactors, and closed containers. The system creates a solid–liquid interface for bacterial colonization and is incapable of being cleaned effectively, posing a risk of cross-contamination. Biofilms pose a strong concern in closed-flow systems. Open systems, on the other hand, represent solid–air and solid–liquid–air interfaces, facilitating bacterial desiccation in a low-*a_w_* food processing environment [33]. Solid–air interfaces represent the direct deposition of microorganisms from the contaminated food to the contact surfaces—whereas solid–liquid–air interfaces are created upon intermittent exposure of the contaminated low-*a_w_* foods to water during wet cleaning and sanitation, thus promoting microbial attachment to the food-contact surfaces [33]. Microorganisms adhering to food-contact surfaces can survive in the starvation phase without the presence of nutrients. This can raise their pathogenicity and subsequently increase their resistance to additional stresses [13,34]. Since production settings have a significant role in cross-contamination, it is essential to follow hygienic zoning within the facilities, as the zones are determined by possible risk sites (Figure 2) [32]. It aids in the identification of niches and harborage areas where prevalent pathogens are found.

**Figure 3:**
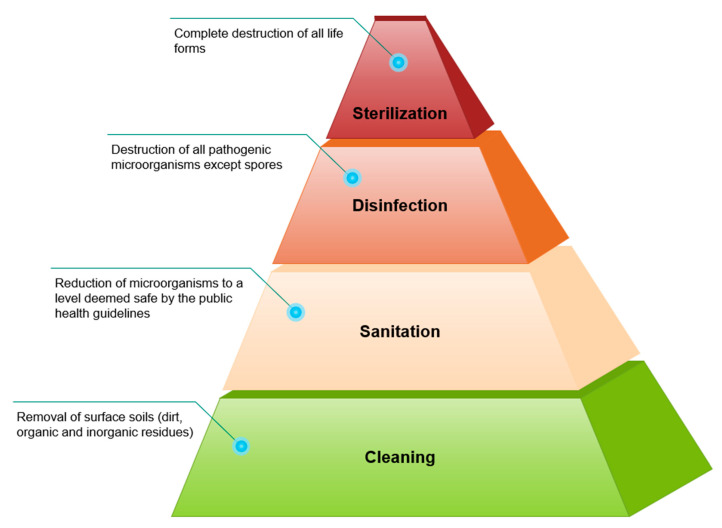
Techniques used to reduce the level of microbial contamination from the food processing facility.

Cleaning, sanitation, disinfection, and sterilization are some of the different approaches that can be taken to lower the level of microbial contamination (Figure 3). Cleaning refers to the removal of surface soils and involves scrubbing, washing, and rinsing food-contact surfaces to get rid of visible contaminants like dust or food particles [36]. Food residues tend to accumulate in cracks, crevices, and irregular contact surfaces; however, they can be easily removed from smooth and nonporous surfaces. Food residue deposits left on food processing equipment after use are often contaminated with microorganisms that thrive off the soil’s nutrients. Usually, soap or another detergent and water are used to remove the soil deposits from the contact surfaces. After cleaning, sanitation and/or disinfection is performed to remove the microorganisms from the contact surfaces [37]. Sanitation is the reduction in microorganisms to a level deemed safe by public health guidelines. Disinfection results in the destruction of all pathogenic microorganisms except spores. Sterilization refers to the destruction of all life forms and is the most lethal of all the processes [38]. There is currently no known antimicrobial agent that is both broadly effective and capable of combining all the necessary properties required for efficient microbial eradication from food-contact surfaces [39]. Hygienic zoning, shown in Figure 2, classifies areas of a facility from most hazardous (Zone 1) to least hazardous (Zone 4) to enforce stricter hygiene standards in high-risk areas and lower them in others [39,40].

A dry food processing facility generally prefers the use of dry methods for cleaning and disinfection. The addition of moisture could potentially cause product decomposition and lump formation. The dry food processing environment, in addition to the processing equipment, must also be kept clean. For instance, the production of dust is a common occurrence in operations that involve the handling of dry materials. Dust clouds can be created when dry ingredients are handled in the normal course of weighing, milling, etc. Pathogens and allergens may be entrapped in process dust, causing cross-contamination [41]. This highlights the significance of preventing and clearing away the dust in dry food-handling areas. Foods manufactured on contaminated equipment compromise food safety and endanger consumer health. Moreover, the abrasive nature of the dust particles could cause equipment deterioration and lubrication issues.

Wet cleaning is performed with oxidizing antimicrobials, such as chlorine-based agents, peracetic acid, hydrogen peroxide, and quaternary ammonium compounds. Generally, wet cleaning occurs after a production run and before the beginning of another production cycle. It can be accomplished with cleaning in place (CIP) or cleaning out of place (COP). Before beginning production, it is essential to thoroughly dry all food-contact surfaces. To control pathogenic microorganisms in food facilities, strict adherence to appropriate hygiene and production control measures is essential [37].

## 2. Factors Influencing the Disinfection Process in Low-Water-Activity Food Processing Environments

Disinfection practices are the cornerstone of any food safety management system. Inadequate disinfection practices may result in foodborne outbreaks [40]. The presence of diverse resident microorganisms on the surfaces of food processing facilities may affect the survival of surface-attached microorganisms [42]. For instance, studies have demonstrated an increased colonization of *L. monocytogenes* in the presence of *Pseudomonas fragi* [43] and *Flavobacterium* spp. [44] on glass coverslips and stainless steel, respectively. Sasahara et al. [43] reported an increased production of exoprotein aids in the entrapment of microorganisms in the multispecies [43]. Moreover, the cell surface hydrophobicity also plays an important role in microbial adhesion on the substratum [45]. On the contrary, certain studies have reported a decreased adhesion of *L. monocytogenes* in the presence of *Staphylococcus* spp. [46]. This indicates a complex interplay of the different bacterial species, cell surface hydrophobicity, and surface characteristics in microbial adhesion on the substratum.

Several factors have been proven to affect bacterial retention on contact surfaces, including the disinfection procedure, bacterial type, presence of organic residues (Table 2 and Table 3), surface chemistry, type of attachment, and surface-free energy of the bacteria and contact surface (Table 4), thereby reducing the efficacy of the disinfection process [33]. The presence of viable cells on biotic and abiotic surfaces contributes to their biotransfer potential [47]. Moreover, studies have demonstrated that surface-dried cells are more resistant to disinfectants than planktonic cells [48].

### 2.1. Biotransfer Potential

Bacterial contamination in the dry food industry can occur either through the air or via food-contact surfaces. Airborne dust and aerosols released during the processing of powdered dry ingredients may contaminate subsequent batches of food manufactured in the same facility [69]. The second type of biotransfer occurs when food-borne pathogens are transmitted from contaminated surfaces to the food itself. The latter form typically occurs when food is contaminated due to contact with unsanitary surfaces or improper handling [69]. There is a risk of biotransfer if exposed surfaces host cells that are alive but not necessarily dividing [33]. The transferability of various microorganisms in both static and dynamic systems has been thoroughly investigated. Dynamic systems represent the moving parts of the equipment, such as slicers, knives, and conveyor belts, whereas the static system comprises the non-moving food-contact surfaces.

The transfer of the surface-attached microorganisms to food is independent of the strain type and the viable counts present on the surfaces. Kusumaningrum et al. [70] observed that *S. aureus* (10^3^–10^5^ CFU/cm^2^) dried at 22 °C at 40–45% RH survived for at least four days on the stainless steel, but when the inoculum load was lowered to 10 CFU/cm^2^, the bacteria reduced to below the detection limit within two days [70]. The study also reported that *S. aureus* dried on stainless steel was more resistant to air-drying than *C. jejuni* and *S. enteritidis* dried under the same conditions. Furthermore, the transfer rates of *S. aureus, C. jejuni*, and *S. enteritidis* from a contaminated sponge to stainless steel were evaluated. Strain type had no significant (*p* = 0.07) effect in increasing the rate of transfer. Moreover, for all the tested strains, increasing the inoculum load had no significant effect (*p* = 0.30) in increasing the transfer rate. This indicates that even though the low inoculum counts could result in higher desiccation reduction, their transfer rates from the contaminated surfaces to the low-*a_w_* foods could potentially be higher, thus posing a food safety risk. Another study by Chen and Snyder [71] evaluated the rate of transfer of *Salmonella* Enteritidis PT30 dried at 20.4 ± 0.8 °C at 48.4 ± 12.8% RH on stainless steel (5.3 ± 0.5 log CFU/coupon), HDPE (5.5 ± 0.5 log CFU/coupon), and rubber (5.4 ± 0.5 log CFU/coupon) to a paper towel [71]. The study reported no significant (*p* > 0.05) effect of the surface material in increasing the rate of transfer of *Salmonella* Enteritidis PT30 to the paper towel.

In addition, the moisture content of food and the type of cell-drying impact the biotransfer potential. Miranda et al. [35] demonstrated that the food’s moisture content significantly influenced the rate at which bacteria transfer from surfaces to the food. For instance, *E. aerogenes* inoculum in TSB and buffer was dried for five hours on stainless steel, tile, wood, and carpet. The study found that a greater transfer rate was observed from all of the indicated surfaces to watermelon (~0.2–97%) regardless of the contact period (1, 5, 30, and 300 s) and that the transfer rate was the lowest for gummy candy (~0.1–62%). Moreover, Hansen and Vogel [47] observed a higher efficiency of transfer of desiccated non-biofilm cells than desiccated biofilm cells of *Listeria monocytogenes* on stainless steel. Transferability occurs as a result of the capillary effect and the possible formation of the liquid bridges between the contact bacterium and the food surface. Furthermore, the study found that rehydrating the desiccated biofilm bacterial cells after contact with salmon led to greater overall water contact, resulting in less transfer than desiccated non-biofilm cells. Limited studies have evaluated the biotransfer efficacy of dried microorganisms on food-contact surfaces to low-*a_w_* foods.

### 2.2. Presence of Persistent and Non-Persistent Strains

Certain strains are capable of surviving in food processing conditions and are repeatedly isolated from similar environments [36,72]. Several outbreaks traced back to the same processing plant have been related to persistent strains. In 2008, the *S*. *enterica* Give outbreak in infant formula was associated with a contaminated production facility [73]. *S.* Poona has been related to another infant formula outbreak that occurred in 2018–2019 due to a contaminated drying tower [74]. A genomic study demonstrated genetic similarity between *S.* Poona isolates collected in 2018–2019 and 2010–2011, proving the persistence of the pathogen through time [74].

Cross-contamination is mostly caused by the occurrence of strains with comparable molecular subtypes, which are associated with persistence [36,75]. Therefore, it is of the utmost importance to comprehend the genetic and physiological behavior of the persistent strains. A multitude of variables, including the temperature and RH of the manufacturing site, play a significant role in microbial persistence. Hiramatsu et al. [56] observed that *Salmonella* serovars dried on paper disks survived for 24 months when stored at 4 °C. However, the bacteria only survived for 70 days when stored at 35 °C. In another study, Chaitiemwong et al. [76] observed a greater reduction in *L. monocytogenes* cells on the conveyor belt at 37 °C and 20% RH with or without antimicrobials than at 10 and 25 °C at 60–75% RH. The results suggested that rapid drying at 37 °C led to a greater decrease, whereas delayed drying at 10 and 25 °C resulted in greater resistance to inactivation, in the presence or absence of antimicrobials. This implies that the environmental conditions of the processing facility must be properly managed to prevent the eventual persistence of microorganisms. Bashir et al. [77] assessed the viability of factory, veterinary, and clinical isolates of *Salmonella* on stainless steel after drying. At 37 °C and 20% RH, all of the isolates were viable for 48 h; however, at 25 °C and 15% RH, they were viable for 22 days. At 10 °C, only *S.* Senftenberg, *S.* Schwarzengrund, and *S.* Typhimurium retained viability for 22 days. It has been reported that higher temperatures cause cellular damage, resulting in a decrease in bacterial survival.

In addition, persistence is also dependent upon the growth phase. Gruzdev et al. [78] observed that stationary *S.* Typhimurium SL 1344 cells dried at 40% RH and 25 °C for 22 h and subsequently stored at 40–45% RH at 4 °C for 8 weeks on polystyrene had a greater desiccation tolerance than those in the early and mid-log phases. This could be due to the potential formation of the Rpos-stress operon [79]. In a similar study, dried *S.* Typhimurium SL 1344 cells grown in LB broth supplemented with 0.5–5% salt conditions showed a higher desiccation tolerance and long-term survival at 4 °C, as compared to cells grown in the absence of salt. It has been reported that salt induces cross-tolerance to a variety of stressors [80].

Certain studies have related the persistence of the strains with their ability to adhere to surfaces [14]. Studies reported that the adherence of persistent poultry plant strains was from 9.1 × 10^2^ cells/cm^2^ to 1.43 × 10^3^ cells/cm^2^ within 1 and 2 h. The adhesion of persistent strains was 2.7–4.6-times greater than that of non-persistent (commonly termed sporadic) strains [81]. Adherence numbers are crucial from a disinfection standpoint. Lunden et al. [81] reported that the shorter contact times could result in a higher attachment of the persistent strains, indicating a reduced effectiveness of the disinfection procedures. The persistence of the organism after sanitation or disinfection suggests that the microorganisms may have created microenvironments and developed resistance to the sub-inhibitory concentration of the disinfectant [82]. It could also have persisted in harborage places such as nooks or crevices [36]. To minimize the persistence and subsequent cross-contamination of pathogens and to limit the associated risks to food safety [1], the dry food industry must adhere to proper disinfection measures.

### 2.3. Presence of Organic Matter

Microbial adhesion to the food processing equipment offers a major risk of pathogen transmission and contributes to foodborne outbreaks [83]. A variety of disinfectants, including quaternary ammonium compounds, amphoteric surfactants, and hypochlorous and alcoholic solutions, are used for sanitation and disinfection. The presence of organic matter can reduce the efficacy of the disinfection procedure [58,61]. It is essential to comprehend the nature of the food soil or organic matter on food-contact surfaces before and after disinfection.

Surface attachment of the bacteria [53,84] and spores [85] in the presence of organic residues provides a protective effect against desiccation [58], disinfection, and heat [86]. The studies on food residues and desiccation resistance are summarized in Table 2 and Table 3. The degree of microbial adhesion will depend on the presence of organic residues on the contact surfaces [87]. Abban et al. [88] observed that *E. coli* attached to residue-free stainless steel at a higher rate (5.18 log cell counts) than it did in the presence of chicken residues (1.5–2.0 log cell counts). However, the cell counts of dried bacteria without organic residues were reduced by 50.8% following rinsing and spraying, whereas they were reduced by only 5.8–16.2% in the presence of chicken residues. Organic residues provide microbial cells with a barrier under desiccated conditions, and as the organic materials are rich in nutrients, they can help in the proliferation of bacteria and can subsequently decrease the disinfectant’s efficacy due to its reduced penetrability [53].

### 2.4. Types of Food-Contact Surfaces

The survival population of microorganisms will vary based on the various food-contact surfaces [33]. Djebbi-Simmons et al. [89] observed greater survival of *S.* Typhimurium on a plastic cutting board (CB) and laminate (LA) than on a stainless-steel cutting board (SS). As a hydrophilic material, SS provides a larger contact area, resulting in the rapid evaporation of the inoculum. LA, on the other hand, is hydrophobic and offers a smaller droplet surface area, resulting in a slower evaporation rate of the bacterial inoculum. Previous studies have attempted to relate the surface characteristics to bacterial adhesion [90]. The surface energy parameters and the contact angle of the various food-contact surfaces are summarized in Table 4. Many factors, such as increased surface area, greater convection mass movement, and decreased shear stress after cleaning, have been associated with higher bacterial adherence on food-contact surfaces [91].

The relationship between roughness values and antibacterial efficacy has been the subject of debate, with some research indicating that the lower the roughness characteristics of the abiotic materials, the greater the microbial inactivation—consequently increasing the hygienic status [92,93]. Kim et al. [94] investigated the relationship between roughness values (mean arithmetic (R_a_) and root mean square (R_q_)) and microbial inactivation utilizing UV-C LEDs. The study reported that dried *S.* Typhimurium, *E. coli* O157:H7, and *L. monocytogenes* were inactivated more effectively on glass, which had the lowest R_a_ (0.0204 ± 0.0005 µm), R_q_ (0.0492 ± 0.0099 µm), and surface contact angle (53.04 ± 1.01°), indicating that it is hydrophilic. Regardless of bacterial species, silicon had the highest R_a_ (0.96 ± 0.02 µm), R_q_ (2.76 ± 0.11 µm), and surface contact angle (118.85 ± 0.03°) and exhibited the lowest inactivation. However, the relationship between the roughness parameters and microbial inactivation remains controversial. Faille et al. [95] explored the relationship between the surface topographies and free energies of various stainless-steel finishes and bacterial adhesion. *Bacillus thuringiensis* was dried with organic food soil (composed of carbohydrates, proteins, fats, and minerals) on stainless steel surfaces with various finishes. They compared various roughness profile characteristics, including the levelling depth (R_P_), arithmetic mean (R_A_), reduced peak height (R_PK_), reduced valley depth (R_VK_), and core roughness depth (R_K_), amongst others. Generally, the roughness factor of 0.8 µm for stainless steel is regarded as applicable for maintaining equipment hygiene in the food industry [88]. The study indicated that a single roughness parameter is inadequate for determining the hygienic status of a surface, as the R_A_ (0.15–1.56 µm) and R_PK_ (0.21–3.49 µm) roughness values differed substantially across the different finished stainless-steel surfaces [95]. Thus, the type of disinfection technique chosen should rely on the type and roughness of the food-contact surface materials.

## 3. Surface Disinfection Challenges Faced by the Dry Food Industry

Severe outbreaks and recalls were reported in low-*a_w_* foods, including *Cronobacter sakazakii* in powdered formula milk (2021), *Salmonella* in peanuts (2009), and chocolate (2022), as well as Shiga toxin-producing *E. coli* in flour (2016) [96]. In addition, *Bacillus cereus*, *Clostridium botulinum*, *Clostridium perfringens*, enterohemorrhagic *E. coli*, *L. monocytogenes*, *S. enterica*, *Staphylococcus aureus*, and *C. sakazakii* were reported as some of the major pathogens involved in dry food processing facilities [97,98]. Some of these microorganisms are known to form biofilms, while *B. cereus, C. botulinum,* and *C. perfringens* are spore formers. Investigations on *B. cereus* and *L. monocytogenes* biofilms found that a single dry sanitization method was not able to eliminate the microbial load [99,100]. Generally, wet sanitization methods are used in food industries; however, due to concerns of sensory properties and the chance of favoring microbial growth, dry sanitization is preferred for dry food processing facilities. Bacteria like *C. sakazakii* were found to be a major threat due to their ability to survive in low-*a_w_* products like infant formula, even causing outbreaks [101]. Another major factor is the residue buildup on contact surfaces and processing machinery; these can be composed of fine powders, as well as oils from nuts (walnuts), which foster microbial growth and fungal growth such as that of fusarium species [98].

Microbial growth and mycotoxin production on the processing surface due to inadequate cleaning, especially in dry materials stuck in small pockets, are real challenges. Moreover, complex equipment designs play a significant role in creating similar scenarios demanding special procedures for cleaning them. Hence, the entrapment of moisture and food residues is a potential cause for creating biological hazards. Also, there is a chance for various cross-contamination occurring through the air during the dry-cleaning process due to the suspended particles in the air. Thus, cleaning surfaces might not be enough to ensure food safety in the processing environment. Overall, biofilms, air quality, and cross-contamination risks in dry food processing facilities need novel methods to address them.

Dry methods are included in the disinfection regime to avoid the potential exposure of moisture in the dry food industry. In contrast, wet disinfection methods are generally incorporated at the end of production processes and are beneficial for the effective removal of food residues from the inaccessible areas of the dry food processing facilities. The disinfection methods are selected based on the product water activity requirements and the process specification steps. For instance, wet disinfection processes are generally incorporated during the initial processing and handling of the dry and wet ingredients. However, the dry disinfection methods are applied at the latter stages of product development to maintain stringent product water activity requirements. In this context, the review categorizes the conventional and novel disinfection technologies into “wet” and “dry” methods to comparatively validate their practicality and antimicrobial effectiveness in assuring the safety of the dry food industry.

## 4. Dry Disinfection Methods for Microbial Inactivation in Dry Food Processing Facilities

The effectiveness of the disinfection process is reduced in the presence of organic and inorganic soils. Consequently, a prior cleaning step is required to remove soiled residues from the food-contact surfaces. Dry or wet procedures may be utilized for cleaning in food processing facilities [102]. The introduction of moisture in dry food industries can be a source of microbial harborage. Thereby, wet cleaning processes are typically unacceptable in facilities that process dry components or low-*a_w_* foods, as the addition of water can promote bacterial adhesion [103]. Dry cleaning methods have gained popularity among food manufacturers in recent years due to an increased risk of food-borne contamination via moisture introduction. Specifically for dry food processing industries, dry disinfection methods are more acceptable as it does not contribute to an increase in moisture content of the environment, which possibly reduces microbial growth potential. Conventional dry-cleaning methods such as brushing, sweeping, scraping, vacuum cleaning, and blowing with compressed air are effective in removing unwanted dust, dirt, and food deposits from the equipment surfaces, walls, ceilings, and floors of the processing plants. However, these techniques are only advantageous for dislodging the “caked-on” residues from the contact surfaces and are not effective in removing bacteria or allergens [37]. Moreover, they are beneficial for cleaning easily accessible locations but may not be able to reach inaccessible areas such as corners and nooks. Inappropriate cleaning methods may also result in equipment deterioration and damage. In addition, they are associated with known constraints, such as the possible generation of dust clouds and the subsequent contamination of the air [37]. Though dry-cleaning cannot guarantee a completely sanitary environment, it can help to reduce product quality loss and food safety hazards. Microbiological assessment of the raw materials used, the process characteristics, and the types of food residues determines the frequency of cleaning. It is crucial to follow appropriate cleaning and disinfection procedures to prevent the spread of food-borne diseases and ensure food safety [85]. This section provides a brief overview of selected conventional and novel dry disinfection methods and their effectiveness in eliminating surface-attached microorganisms in the dry food industry. The antimicrobial mechanisms of the various dry disinfection methods are discussed in the following sections in more detail.

### 4.1. Conventional Dry Disinfection Methods

#### 4.1.1. Isopropyl Alcohol-Quaternary Ammonium-Based Disinfectants

**Figure 4:**
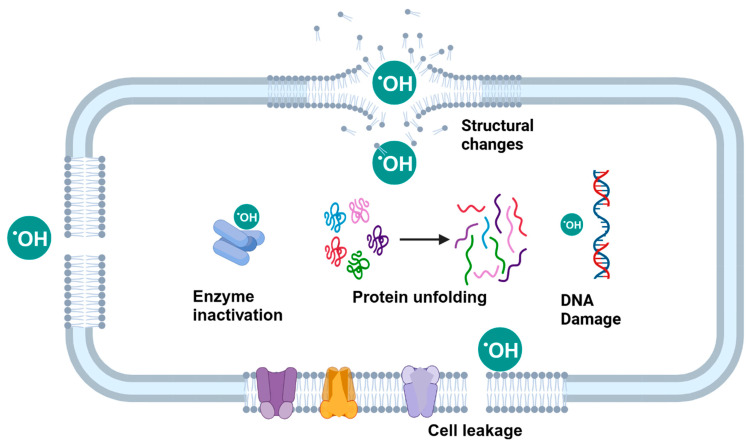
Disinfection mechanism of alcohol-based disinfectants. Created with BioRender.com.

**Figure 5:**
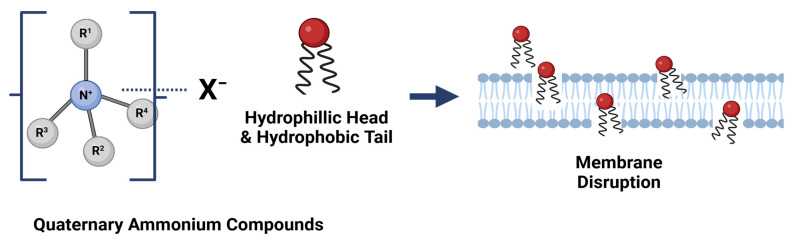
Disinfection mechanism of quaternary ammonium compounds. Created with BioRender.com.

Traditional dry-cleaning techniques, including brushing and scraping, are not appropriate for all low-*a_w_* foods, such as nut products with a high fat content and viscosity, contributing to their better adhesion to food-contact surfaces [104]. Alcohols are generally used for surface disinfection due to their antimicrobial properties; they permeate the cell membrane and denature proteins, resulting in the lysis of the cells. Alcohol present in disinfectants initially interacts with the cell membrane, which consists of a lipid bilayer. This interaction induces structural alterations in the membrane, causing it to expand laterally while contracting vertically. These changes ultimately result in membrane failure (Figure 4) [105]. Quaternary ammonium compounds (QUAT) possess bactericidal and fungicidal properties. The structure of QUAT features a positively charged nitrogen atom bonded to either four substituents or three substituents with one double bond [106]. These are positively charged cations that are attracted to negatively charged cellular components. The mode of action of QUAT involves the process of perturbation that results in the disruption of the membrane bilayers by the alkyl chains and the disruption of charge distribution of the membrane by the charged nitrogen. This results in the progressive leakage of cytoplasmic components out of the cell and, finally, cell lysis (Figure 5) [107].

It is possible to improve microbial inactivation efficacy by combining QUAT and alcohols [108]. Isopropyl alcohol-quaternary ammonium (IPAQUAT) formulations have a relatively modest concentration of quaternary ammonium compounds along with a varying concentration of isopropyl alcohol. Alcohol-quaternary formulations are non-corrosive and suitable for disinfecting non-porous surfaces [103,104]. Moreover, carbon dioxide (CO_2_) can be used as a propellant for IPAQUAT spray application. CO_2_ displaces oxygen and renders it non-flammable, hence eliminating the potential for a fire hazard [108]. Kane et al. [103] studied the effectiveness of IPAQUAT-CO_2_ (200 ppm, 58.6% isopropyl alcohol) spray for eliminating *Salmonella* cells dried (16–18 h) on stainless steel. An IPAQUAT-CO_2_ exposure for 30 s resulted in a reduction in the dried *Salmonella* cells by 6.18 log CFU/25 cm^2^. However, *Salmonella* dried in the presence of breadcrumbs was reduced to >6.02 log CFU/25 cm^2^ after 30 s of IPAQUAT treatment.

The IPAQUAT formulation does not require water and has a high evaporation rate, making it appropriate for disinfecting dry food processing plants. The IPAQUAT’s active quaternary ammonium component generates an antimicrobial film for surface disinfection. The concentration of the QUATs can be controlled to avoid an excessive buildup of residual film on the contact surfaces [103]. Before the application of IPAQUAT, food soil must be eliminated through cleaning [37]. Grasso et al. [104] followed a two-step cleaning and sanitation procedure to decontaminate *Salmonella*-infected (7.4 ± 0.4 log CFU/g) peanut butter stainless steel processing equipment. Hot oil cleaning alone at 90 °C for 2 h resulted in a 0.25 ± 1.12 log CFU/cm^2^ *Salmonella* survival count. However, a two-step combination of cleaning using hot oil (90° C for two hours), followed by disinfecting using isopropanol with quaternary ammonium compounds (60% for one hour) resulted in a 5-log reduction in *Salmonella* from the various sampling sites of the contaminated equipment. Moreover, the disinfection efficacy of IPAQUAT will vary based on the type of surface and combination of treatments. Du et al. [109] studied the effectiveness of different cleaning and disinfection treatments in reducing the aerobic load from the typical surfaces found in the almond huller–sheller facilities. A combination treatment of blowing air for 30 s followed by an IPAQUAT (200 ppm, 58.6% isopropyl alcohol) spraying for 60 s resulted in a higher reduction in the aerobic counts by 2.1 and 3.6 log CFU/cm^2^ from the conveyor belt and galvanized steel, respectively. Limited research has been conducted using IPAQUAT for the eradication of microorganisms from the dry food industry.

There are numerous challenges associated with the use of alcohol-based disinfectants and QUAT for dry disinfection. Although these disinfectants are effective in reducing microbial populations, their application often leads to the formation of significant amounts of byproducts, particularly when used directly. These byproducts, as noted by [110], can be harmful and toxic to human health, raising considerable public health concerns. Furthermore, Mahapatra et al. [111] have highlighted additional issues, including the potential for microbial resistance in certain strains, alongside various health risks associated with prolonged exposure to QUAT disinfectants. A study by Yeung et al. [112] revealed that several alcohol-resistant strains have emerged in recent years, posing challenges to their removal and increasing risks to food safety. Efforts to reduce the concentration of these compounds to minimize toxicity have the unintended consequence of diminishing their disinfecting efficacy, creating a complex balance between safety and effectiveness in disinfection practices.

#### 4.1.2. Fumigation with Gaseous Antimicrobials

Food processing facilities use vapor, fog, and gaseous fumigation techniques to effectively eradicate microorganisms and ensure food safety [113]. Vapor consists of a single molecule of gas and may or may not be visible. In contrast, fog consists of tiny, condensed forms of water droplets suspended in the air and forms a dense aerosol. Droplets usually accumulate as a thin film of condensation on the exposed parts of the equipment’s surface. Inadequate dispersal or a “shadowing effect” caused by equipment that blocks the fumigants’ path could be the biggest drawback to using vapor or fog fumigants [113]. Secondly, there is an influence of gravity, as the vapor or fog may not be able to travel a greater distance [113]. The third class consists of gaseous fumigants. Gases fill an entire space and disperse more quickly, making it simpler to disinfect the ceilings, corners, cracks, and crevices of a food processing facility. Chemical gas fumigants can be divided into oxidizing and alkylating agents. The most common alkylating agents are methyl bromide, ethylene oxide, and propylene oxide, whereas oxidizing agents include chlorine dioxide and ozone [113]. Methyl bromide functions as a fungicide, insecticide, and pesticide in controlling the insects and pests from the bulk stored commodities [114,115]. The bromine released during its decomposition is known to disrupt the natural cyclical processes that contribute to the stratospheric ozone layer’s depletion [116]. Nevertheless, it is a potent carcinogen and was banned as part of the Montreal Protocol in 2005 [114]. Propylene oxide (PPO; C_3_H_6_O) is a safer and more eco-friendly alternative to methyl bromide [117]. It functions as a fumigant and microbial sterilant to eradicate microflora such as bacteria, mold, and yeast [118] from low-*a_w_* foods such as processed spices, cocoa, dried foods [119], and processed tree nuts [118,120]. PPO residues in foods are converted to non-hazardous propylene glycol [121]. The FDA and the EPA have permitted the use of PPO as a gas sterilant in conjunction with carbon dioxide for prevention of the microbial contamination and as an insect infestation of spices and tree nuts [117,122]. PPO is also used for fumigating post-harvest agricultural products [123].

Ethylene oxide (ETO) is another widely used type of gas fumigant. ETO fumigation is a dry, non-thermal method and is utilized for the decontamination of medical devices and low-*a_w_* products such as spices [124,125]. The European Commission’s maximum residue level (MRL) regulation, No. 396/2005, includes ETO as a pesticide [126]. It has been classified as a category 1B carcinogen [127] due to its ability to produce carcinogenic derivatives like ethyl glycol and 2-chloroethanol and has therefore been prohibited in the European Union since 1991 [128]. Its antibacterial, antifungal, and antiviral effectiveness has led to its widespread use in countries such as Canada, the United States, and India [126]. After ethylene oxide (ETO) fumigation, food must be aerated for at least 24 h to remove all residues of the gas [129]. The ETO residue left in foods after improper fumigation interacts with inorganic compounds and leads to the production of hazardous byproducts such as ethylene chlorohydrin, ethylene glycol, and ethylene bromohydrin [129]. ETO and ethylene chlorohydrin levels in spices following fumigation have been advised not to exceed 7 and 940 ppm, respectively [130]. Despite the widespread use of ETO gas fumigation for sterilizing spices, vegetables, and beverages, concerns about its carcinogenic properties have prevented any attempts to utilize it for disinfecting food processing industries.

Fog-based fumigation techniques require extensive research on their utilization as a sanitation agent in the dry food industry. Currently, very few studies have reported their effectiveness for microbial inactivation on food-contact surfaces, thus limiting their discussion in this review. Fumigation with oxidizing agents such as chlorine dioxide and ozone is commonly used to disinfect food processing facilities, and the next section provides a concise overview of these technologies.

#### 4.1.3. Chlorine Dioxide Gas Fumigation

**Figure 6:**
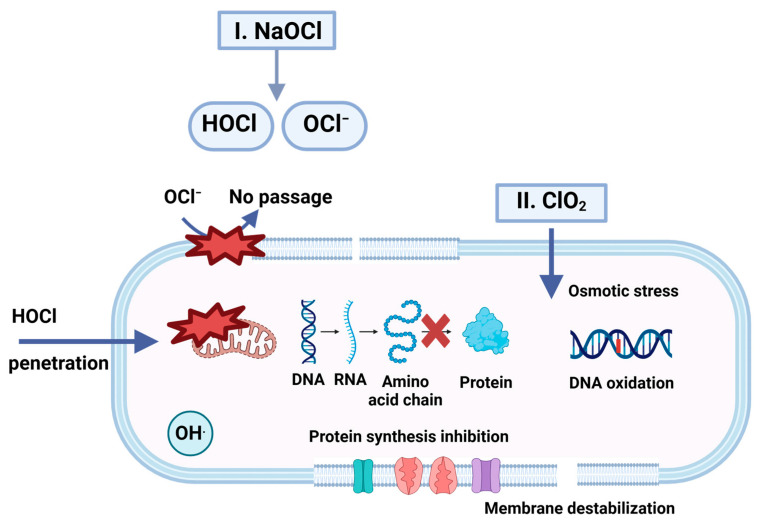
Disinfection mechanism of chlorine dioxide gas and sodium hypochlorite. Created with BioRender.com.

Chlorine dioxide (ClO_2_) gas treatment is a non-thermal method used for equipment sanitation [131]. It is a synthetic reddish-yellow gas [132,133] that functions as a potent biocidal disinfectant [134] by removing an electron from electron-rich sites on biological molecules and subsequently reducing to ClO_2_^−^. It penetrates the bacterial cell membrane and causes lipid peroxidation, protein denaturation, and DNA damage (Figure 6). The oxidation capacity of ClO_2_ is 2.5-times greater than that of liquid chlorine [135]. Extensive research has been undertaken on the use of ClO_2_ gas to disinfect food products and remove biofilms from contact surfaces [136]. It works over a broad pH range (3.0–8.0) [133] and prevents the formation of chloramines and other halogenated organic compounds [137]. Moreover, its fast action and on-site production [138] make it appropriate for use in dry food industry disinfection. The investigations on the usage of ClO_2_ gas for the dry microorganisms on food-contact surfaces are summarized in Table 5.

It is generally recommended that the equipment used in dry procedures should not be wet-cleaned except under specific conditions. ClO_2_ in gaseous form penetrates uneven surfaces more effectively than aqueous chlorine dioxide [140]. The application of aqueous chlorine in the dry food industry is not preferred as it can introduce unwanted moisture if it is not drained properly, resulting in microbial growth. Gaseous ClO_2_ has greater diffusibility and produces fewer toxic residues, leading to a greater reduction in pathogenic microorganisms [141]. Amino acids are oxidized by ClO_2_ according to pseudo-first-order kinetics, which impedes protein synthesis. Furthermore, the oxidative stress coupled with respiration inhibition causes cell destabilization [133].

The mechanisms involved in the inactivation of microbes by ClO_2_ are oxidation, cell rupture, and total cell damage. ClO_2_ oxidizes the cell wall by taking away electrons from the cell wall and membranes, which disrupts the microorganism’s vital structure. Furthermore, it reacts with amino acids in the cytoplasm, which ultimately creates an imbalance within the cell. Hence, the cell structure collapses, and microorganisms can no longer survive [142,143]. In integral membrane proteins, tryptophan residues are assumed to have unique functions, anchoring transmembrane alpha-helices into the lipid bilayer [144]. The accessibility of the tryptophan residue in the peptide or protein, as well as the nearby amino acid residues, determines the reaction rate of ClO_2_ with proteins [145].

The effectiveness of ClO_2_ gas depends on intrinsic parameters such as roughness [93] and hydrophobicity, as well as extrinsic parameters such as temperature [131], contact time, and gas concentration [146]. Moreover, pre-humidification and the state (wet and dry) of microbial culture are crucial to the inactivation efficacy of ClO_2_ gas [132]. In a study conducted by Yeap et al. [134], stainless-steel coupons were contaminated with murine norovirus 1 (MNV-1). Before ClO_2_ treatment, coupons were preconditioned at 85% RH at 25 °C for 10 min. ClO_2_ treatment at 2.5 mg/L for 2 min resulted in a 3-log reduction in the virus. Moreover, no infectious virus was recovered when the dosage was increased to 4 mg/L. Morino et al. [147] investigated the efficacy of a low concentration of ClO_2_ (0.08 ppm) at 45–55% against feline calicivirus (FCV) in the dry and wet states on glass. The wet condition resulted in a greater reduction of >6 logs after 6 h, while the dry state resulted in a smaller reduction of <2 logs after 48 h. Pre-humidification of the virus is preferred to increase the solubility of the ClO_2_ gas, thus resulting in an increased denaturation of the viral capsid proteins (tyrosine, cysteine, and tryptophan amino acids) [148].

In another study conducted by Morino et al. [132], ClO_2_ at 0.05 ppmv for 4 h was used to treat suspension cultures of *E. coli* and *S. aureus* on glass surfaces. The reduction in *E. coli* (>5.0-log reduction) was greater than that of *S. aureus* (>2-log reduction). The application of chlorine dioxide with spray chilling resulted in a 3-log reduction against *E-coli* and *Salmonella.*

The surface characteristics influence the effectiveness of ClO_2_ gas against microbial inactivation [93]. In general, hydrophilic surfaces allow for a more uniform attachment and distribution of microorganisms than hydrophobic surfaces, resulting in a higher level of inactivation. Park et al. [93] evaluated the efficacy of a 15 min ClO_2_ treatment at 20 ppmv against *Salmonella* Typhimurium on a variety of food-contact surfaces. The treatment of the dehydrated cells was conducted under humidified conditions (90% RH). Compared to hydrophobic materials such as silicon (0.96 ± 0.45 log CFU/cm^2^) and rubber (1.90 ± 0.32 log CFU/cm^2^), the high hydrophilicity of glass resulted in a greater reduction of >6.76 log. The research indicated that the dosage or duration of ClO_2_ treatment should be increased under dry conditions to obtain a greater microbial reduction. Fu et al. [149] showed that ClO_2_ gas fumigation can inhibit spore formation in spices. They noted that *B. cinerea* cells treated with 50 μmol L^−1^ of ClO_2_ exhibited signs of shrinkage, pitting, and fragmentation, along with the adhesion and aggregation of damaged cells. Furthermore, it was reported that disinfection effectiveness highly depends on the concentration used. ClO_2_ gas has several drawbacks, including its instability at higher concentrations, its difficulties in handling and transportation, and its high cost for on-site generation and mixing [133].

#### 4.1.4. Ozone Gas Fumigation

Ozone is a colorless, highly reactive gas with strong antioxidant and biocidal properties [150]. It is composed of three single oxygen atoms covalently linked together [151]. Ozone can be produced photochemically by bombarding an oxygen-containing gas with ultraviolet light or passing air through a corona discharge, utilizing a high-energy electric field to trigger the creation of free radicals [152]. Ozone is an unstable oxygen allotrope with a half-life of nanoseconds [153]. It auto decomposes, produces oxygen, and equilibrates with air, so it neither leaves behind residual byproducts [154] nor increases the water salinity. In addition, it minimizes the cost of sewage disposal by oxidizing organic material and facilitating its biodegradation, hence minimizing surface deterioration and environmental impact [153]. Ozone can be administered in either its gaseous or aqueous (ozonated water) forms [154]. Gaseous ozone provides an alternative sanitation technology to chemical sanitizers. With an oxidation–reduction potential (ORP) of +2.07 V, ozone is a suitable alternative to chlorine’s lower ORP of +1.49 V [155]. Numerous studies have highlighted the advantages of gaseous ozone over conventional disinfection procedures, as it does not include water and hence eliminates the need for rinsing [153]. It can be effectively used to inactivate the airborne microorganisms generated during the handling of dust clouds. It is spreadable and accessible, and it can easily penetrate the cracks and crevices of the contact surface [156]. Moreover, ozone can expand and take up the volume of the whole room, making it advantageous over other surface treatments such as UV light [151]. Nevertheless, the efficiency of ozone as a disinfectant is much enhanced at higher humidity levels [155], and the magnitude of the bactericidal activity will be reduced at lower humidity levels. UV irradiation can increase the disinfection kinetics of ozone under high humidity conditions. The subsequent interaction of ozone with water results in the production of highly reactive hydroxyl radicals, thus increasing the decontamination effectiveness [155].

The surface disinfection efficiency of the ozone is dependent on pH, temperature, ozone concentration, treatment time, surface type, and the presence of organic residues [151]. It can be generated on-site and requires no additional handling and storage [153]. Generally, ozone at a higher concentration is more effective for antimicrobial action [157]. However, the major disadvantage of ozone is its potential toxicity, which can have detrimental impacts on contact surfaces and human health [158]. In its gaseous form, ozone is more harmful than when it is present in water. The inhalation of ozone might cause peripheral vasoconstriction [159]. The recommended threshold for ozone exposure is 0.1 ppm [160]. Degassing is necessary before entry or human exposure, and adequate implementation or application procedures are required [156]. Therefore, sufficient time must be allowed for ozone breakdown before entry.

**Figure 7:**
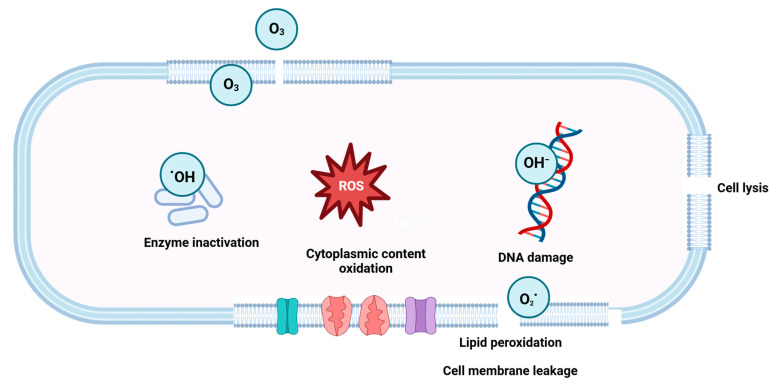
Disinfection mechanism of ozone. Created with BioRender.com.

Ozone has been the subject of numerous investigations due to its broad spectrum of antimicrobial potential against bacteria [157], viruses [156,158], and mycotoxin degradation [154]. Ozone has a strong penetrating power and destroys cells by removing hydrogen atoms from carbon–carbon bonds, hence causing metabolic interference and cell lysis. Furthermore, the generation of reactive species upon ozone decomposition affects the cellular metabolism by targeting membrane glycolipids and glycoproteins and further oxidizes protein and lipid components, resulting in cellular leakage (Figure 7) [157]. The decomposition of O_3_ involves two actions.

Fast action:

(1) $$O_{3}\to O_{2}\;+\;O$$

Slow action:

(2) $$O^{\cdot }\;+\;O_{3}\to 2O_{2}$$

From the first action (Equation (1)), singlet oxygen species are generated, which react with the bilayer lipid and oxidize it [161]. As Reaction 1 is quick, the oxidizing reaction swiftly takes place, which leads to higher penetration by ozone. Xue et al. [162] showed that ozone exhibits a higher oxidizing potential (2.07 V) than other oxidizers such as hydrogen peroxide (1.78 V), chlorine gas (1.36 V), and oxygen (1.23 V). Other than the oxidization of the membrane and cell wall, which leads to cell wall leakage, O_3_ can disrupt the DNA structure by breaking the double-bond formation within the structure [162].

Kim et al. [163] investigated the efficacy of ozone against airborne and surface-attached *Pseudomonas aeruginosa*. Ozone (2 ppm) exposure for 2 h resulted in a 2-log reduction in the number of cells of both types. However, whether ozone is more efficient in the aqueous or gaseous phase is quite debatable. Several studies have been conducted on different produce and microbes with ozone [164,165,166]. These studies indicate that the application and efficiency of ozone highly depends on the type of produce, the initial load, and susceptibility of the particular microbes to ozone.

The gas-phase application of ozone can be used for surface disinfection in the dry food industry. Dried cells of *Escherichia coli*, *Shigella liquefaciens*, *Listeria innocua*, *Rhodotorula rubra*, and *Staphylococcus aureus* on stainless steel had their microbiological viability reduced from 7.56 to 2.41 log values after being treated with ozone at 2 ppm for 4 h at 77% RH [150]. Bailey et al. [167] reported that *Micrococcus luteus* on stainless steel was reduced by 2–3 logs after being treated with 2 ppm ozone for 1 h at 50% RH. While increasing the humidity levels has been shown to boost antibacterial efficacy, it introduces unwanted moisture into the dry food manufacturing sector. Candia et al. [157] demonstrated the efficacy of the low-concentration gaseous ozone treatment (1.07 mg m^−3^) in inactivating dried cells of *Listeria monocytogenes*, *E. coli*, and *S. aureus* on stainless steel, glass, polystyrene, and polypropylene and observed no viable cells after 6 days of treatment at 4 °C. Dubuis et al. [168] discovered that gaseous ozone treatment at 0.05 ppmv for 30 min led to the inactivation of the SARS-CoV-2 virus by causing damage to the viral capsid protein.

Ozone is approved as an antimicrobial addition by the US Food and Drug Administration (FDA) and has GRAS (generally regarded as safe) affirmation for use in food-contact applications [169]. Moreover, the European Union has authorized the use of ozone for the treatment of wastewater [150].

### 4.2. Novel Dry Disinfection Technologies

#### 4.2.1. Superheated Steam

The use of superheated steam (SHS) for surface disinfection has only been the subject of a small number of studies despite its widespread investigation as a dehydration method [85]. SHS is a promising technology that can be implemented as a dry disinfection tool in the low-*a_w_* food processing industry [170]. SHS is produced by raising the temperature of the saturated steam by supplying sensible heat at a constant pressure [85]. SHS can inactivate vegetative cells, spores, and biofilms [171].

SHS is non-polluting and does not require the use of chemicals. The thermal energy of SHS is greater than the equivalent volume of water at a given temperature. It is advantageous for surface disinfection, as it offers a higher heat transfer to the contact surface and, upon condensation, raises the temperature of the contact surfaces, thereby providing a better latent heat transfer [171]. In addition, it is safe and results in a lower quality loss [172,173]. The distinctive advantage of SHS over saturated steam is that a slight decrease in temperature does not result in condensation and will prevent the unwanted deposition of water on the food-contact surface [174]. Moreover, SHS has a higher efficiency and better penetration into cracks and crevices and does not produce toxic byproducts.

**Figure 8:**
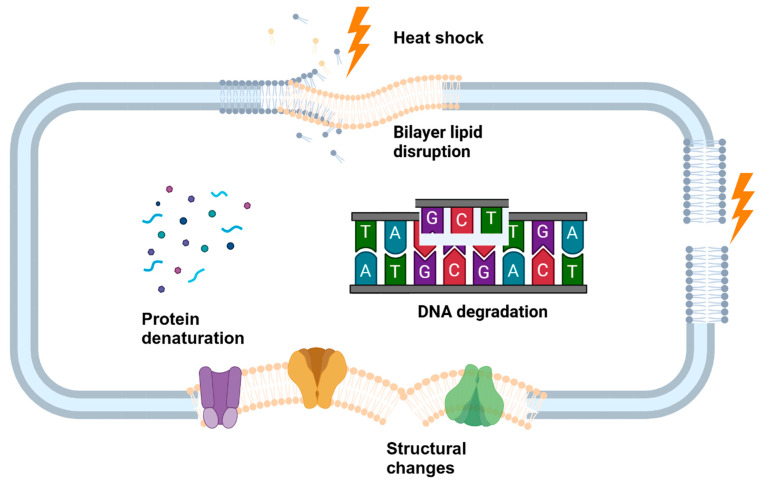
Disinfection mechanism of superheated steam. Created with BioRender.com.

High temperature denatures the cytoplasmic protein and induces membrane fluidization, resulting in cell death (Figure 8) [37]. Ban et al. [175] reported that SHS treatment of 200 °C for 10 s reduced the log counts to below the detection limit of 1.48 log CFU/cm^2^, of the *Salmonella* biofilms formed on the stainless steel. It has been well documented that bacterial exposure to sub-lethal temperatures induces a heat shock response and regulates the synthesis of various heat shock proteins (HSPs) [176,177]. These proteins help to rebind the proteins and sustain heat shocks. However, the high temperature of superheated steam can make the HSP non-functional by structural alterations, introducing a weak point in the cell’s defense mechanism [178]. Proteins may unfold or misfold because of heat shocks and other unfavorable development conditions, interfering with many cellular processes [178]. Eventually, the cells stop functioning, leading to cell lysis and cell death. A study by Ban et al. [179] evaluated the transcriptional response of *Salmonella* Typhimurium biofilms after SHS treatment at 200 °C for shorter intervals of 1, 3, 5, 10, and 20 s. The study observed an upregulation of the genes encoding several HSPs after 1 s of SHS treatment. Nevertheless, studies related to the transcriptional response of bacteria with superheated steam are limited and demand a detailed investigation.

Kim et al. [171] reported that SHS treatment (250 °C for 1 min) reduced *B. cereus* inoculated on stainless steel by 0.68 ± 0.27 log CFU. At temperatures > 250 °C, spores were eliminated within 1 min. The combined efficacy of UV-C (15 min) followed by SHS (250 °C, 1 min) offered a synergistic effect and reduced spores by 2.30 ± 0.50 log CFU.

The organic residues on the contact surfaces must be removed by thorough cleaning. Inadequate cleaning before SHS exposure might result in baked-on residues. Kim et al. [171] investigated the effect of food composition on the antimicrobial effectiveness of SHS against *B. cereus* spores. SHS (161 ± 1° C) resulted in lower *D*-values (46.53 ± 4.48 s) for low-fat peanut butter (6% fat and 55% moisture) and a higher *D*-value (79.21 ± 14.87 s) for high-fat peanut butter (43% fat, 10% moisture). On the contrary, non-fat dry milk and whole milk powder had lower *D*-values of 24.73 ± 6.78 s and 34.38 ± 20.08 s, respectively. Kim et al. [171] further investigated the efficacy of SHS treatment for removing food residues from aluminum foil. The study showed that the ease of removing food residues depends on the food composition and the duration of SHS exposure. SHS (161 ± 1 °C, 30 s) resulted in a residual weight removal of 99.07 ± 0.15% of peanut butter (50% fat, 18% carbohydrate) as compared to the 36.22 ± 2.88% residual weight removal of the non-fat dry milk (0% fat, 52% carbohydrate). Due to the impact of food components on surface adherence, SHS treatments must be preceded by adequate cleaning. Park et al. [85] studied the efficacy of the SHS treatment in inactivating *Geobacillus stearothermophilus* inoculated along with wheat flour on various food-contact surfaces (stainless steel, rubber, and concrete). The study reported a higher heat transfer and temperature increase in the concrete, which resulted in rapid drying of the wheat flour, resulting in a lesser reduction in *G*. *stearothermophilus*. On the contrary, irrespective of the treatment temperature, a higher reduction in *G. stearothermophilus* was observed on the stainless steel. The study also reported that increasing the RH of superheated steam can result in a higher reduction. The study reported that the higher vapor diffusion in the porous concrete resulted in the subsequent drying of the wheat flour. Moreover, the high thermal diffusivity of steel (3.54 × 10^−6^ m^2^/s–3.92 10^−6^ m^2^/s) resulted in heat dissipation by working as a heat sink, thereby requiring longer treatment times to reach the target temperature [85] There is a lack of research into the potential of supersaturated steam for disinfection application in the dry food industry.

#### 4.2.2. UV Light Disinfection

UV has a broad electromagnetic spectrum ranging from 100 to 400 nm. It can be divided into UV-A, UV-B, UV-C, and UV-vacuum categories. UV-C has the maximum germicidal potential; it causes the alteration of DNA/RNA at 254 nm [180]. The US Food and Drug Administration has approved ultraviolet light at 254 nm as a non-thermal decontamination technology for disinfecting food-contact surfaces [181,182] and for treating surface microorganisms on food products [183]. It is also commonly utilized for its ability to disinfect the air and water.

UV triggers the transition from the ground state to the excited state of a molecule. Its mode of action has been attributed to numerous mechanisms, including the following [180].

(a) Fluorescence, wherein the molecule returns to its ground state by emitting a photon.
(b) Phosphorescence, which indicates that the molecule will maintain its excited state.
(c) Internal conversion, in which heat is lost as the medium returns to its initial state.
(d) Photochemical reaction, involving chemical conversion by altering the chemical structure of the molecules such as DNA/RNA.

**Figure 9:**
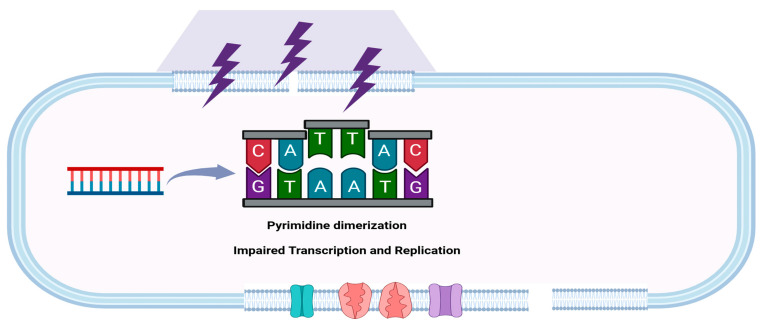
Disinfection mechanism of ultraviolet light. Created with BioRender.com.

UV has been primarily linked to the photochemical modification of pyrimidine bases, which leads to the formation of dimers between successive pyrimidines in a DNA strand [180] and results in the formation of a DNA bend, which in turn inhibits the action of DNA polymerase, thereby blocking transcription and replication and causing cell death (Figure 9) [184]. It inactivates the pathogen, rendering it incapable of reproduction [185,186].

$$2\;Thymine\;\frac{UV}{--}\;\to \mathrm{Cyclobutane}\;\mathrm{thymine}\;\mathrm{dimer}$$

$$Thymine\text{-}Thymine\;\frac{UV}{--}\;\to \;TT\text{-}dimer$$

Protein also undergoes some alterations because UV light can act as a catalyst and change the structure of amino acids, e.g.,

$$Cysteine\frac{UV}{--}\;\to \;Methoinine\;sulphide\;or\;cysteine\;sulphide\;$$

The structural and chemical characteristics of amino acids change, affecting their cell functions. In addition, UV also generates reactive species during its interactions with the surrounding atmosphere. The most common reactive species generated by UV are superoxide (O_2_·^−^), hydrogen peroxide (H_2_O_2_), and hydroxyl radicals (·OH). The generation of reactive oxygen species (ROS) can cause oxidative stress, as these species are highly reactive [57,186]. Reactive species react with DNA, protein, and cytoplasm, creating a structural imbalance within the cell. The structural imbalance affects the life cycle of the cell, leading to leakage through the damaged cell wall, DNA rupture, and mutation in the cell. Additionally, UV can activate stress responses in the cell to repair damaged DNA and other areas, delaying mitosis [187].

In addition, UV application has many benefits, including the absence of odors and harmful residues, low costs, ease of use, and lack of regulatory constraints and limits [188]. It is chemical- and heat-free and relatively inexpensive [180]. Table 6 provides a summary of the studies demonstrating UV light’s effectiveness in inactivating surface-dried microorganisms from food-contact surfaces.

UV disinfection has several disadvantages, including low penetrability and a shadowing effect [28]. Conventional UV lights utilize UV mercury lamps, but they operate at high voltages and are frequently linked to the formation of ozone, which is hazardous to human health. UV-LEDs are, therefore, becoming increasingly popular. Calle et al. [184] examined the effectiveness of UV-C LED (250–280 nm) at 2 mW/cm^2^ (50%) and 4 mW/cm^2^ (100%) for 60 s against *Salmonella* attached to stainless steel and high-density polyethylene (HDPE). The results from the study showed that treatment with 50% irradiance resulted in a 1.97 log CFU/cm^2^ and 1.25 log CFU/cm^2^ reduction in *Salmonella* on stainless steel and HDPE, respectively. Increasing the irradiance to 100%, they observed a higher reduction in *Salmonella* by 3.48 log CFU/cm^2^ and 1.77 log CFU/cm^2^ on stainless steel and HDPE, respectively. The antibacterial efficacy of UV light against foodborne microorganisms adhered to the food residues on contact surfaces has been widely investigated. Kuda et al. [84] studied the antibacterial effectiveness of UV-C (254 nm) on dried suspensions of *Salmonella* Typhimurium, as well as *Staphylococcus aureus* soiled with 1.5–15% *w*/*v* egg albumen, 1.5–15% yolk, or 3.0–30% whole egg solutions on glass surfaces. It was observed that the food sediments had a protective effect on the bacteria against drying and UV-C treatment.

Certain microorganisms, including *E. coli*, *Salmonella,* and *Shigella dysenteriae*, can repair UV-induced DNA damage by photoreactivation. The photolyase enzyme reverses the thymine dimerization mutation in the presence of sunlight. Additionally, certain species are capable of independent repair without light; endonucleases can cleave the altered DNA, and polymerase can then synthesize a new DNA fragment. Finally, ligases can join these fragments together [181].

#### 4.2.3. Cold Plasma

Cold plasma, also referred to as the fourth state of matter [193], is an emerging non-thermal technology used for the disinfection of bacteria [189], viruses [194], and spores [195]. Gas, when subjected to a source of energy, dissipates and creates free radicals, reactive oxygen (O, ^1^O_2_, OH●, O_3_, and H_2_O_2_) and nitrogen species (N, NO_3_^−^, NO_2_^−^ NO●, ONOO^−^, and HNO_3_), electrons, photons, and neutral and charged particles. They are primarily produced through the collision of high-energy electrons with heavy particles such as atoms, molecules, and ions [196,197].

**Figure 10:**
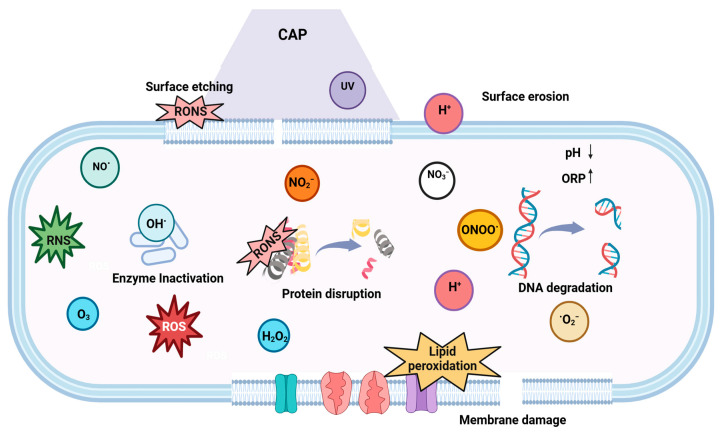
Disinfection mechanism of cold plasma. Created with BioRender.com.

The antimicrobial mechanism of plasma involves multiple mechanisms, including thymine dimerization, the inhibition of replication in response to UV-induced DNA damage, and surface etching of the cell membrane due to the accumulation of excessive charge [196,198]. It leads to the removal of the H atoms, resulting in the cleavage of C–O, C–C, and C–N bonds and the production of C = O bonds [199]. In addition, ROS can disrupt the peptidoglycan linkages of the outer membrane, leading to lipid peroxidation, protein denaturation, and DNA damage, thereby contributing to cell death [197]. ROS-generated oxidative stress additionally leads to the inactivation of microorganisms (Figure 10) [198].

Peptidoglycan cross-link peptide + ROS → degraded peptide bonds

Lipid peroxidation is a major detrimental process in cells in which ROS damage unsaturated fatty acids in the walls of cells, notably the outer membranes of Gram-negative bacteria [80,170].

lipid molecule (L) +ROS → lipid radical (L·)

lipid radical (L·) + O_2_ → lipid peroxyl radical (LOO·)

lipid peroxyl radical (LOO·) +ROS → lipid hydroperoxide (LOOH) + lipid radical (L·)

However, the type of reactive species generated is dependent on the gas used for plasma generation [200]. The main reactive species in O_2_-based plasma are atomic oxygen (O_2_), ozone (O_3_), singlet oxygen (^1^O_2_), superoxide anion (O_2_^−^), and hydroxyl radicals (•OH); the main reactive species in N_2_-based plasma are nitric oxide (NO), nitrogen dioxide (NO_2_), peroxynitrite (ONOO^−^), nitrate (NO_3_^−^), and nitrite (NO_2_^−^); the main reactive species in air-based plasma are combinations of reactive oxygen and nitrogen species (RONS); and the main reactive species in Ar- or He-based plasma are excited He or Ar radicals and hydroxyl radicals.

Microbial inactivation will differ depending on the plasma source, such as dielectric barrier discharge (DBD), plasma jet, gliding arc, microwave-based discharge, corona discharge, or glow discharge [201]. DBD is appropriate for surface disinfection since it is a direct treatment that can disinfect larger surfaces, is inexpensive and scalable, and can be operated under ambient conditions [193]. Treatment with DBD plasma for 340 s reduced *L. innocua* inoculated on spinning knives by more than >5 logs [199].

Several other parameters, including voltage, frequency, flow rate, structural properties of the contact surface, and treatment time, influence the effectiveness of cold plasma in inactivating foodborne microorganisms [202]. The inactivation efficacy of cold plasma reduces as the distance between the plasma source and the sample increases. *E. coli* being surface-dried for one hour on polyvinyl chloride followed by five minutes of air DBD plasma treatment at spacings of 3, 4, and 5 cm correspondingly led to reductions in the germicidal efficacy of 99.99%, 99.1%, and 98.9%, respectively [203]. The efficacy of this technique also depends on the type of soil accumulated on the contact surfaces [204]. Gonzalez et al. [201] examined the inactivation efficacy of the piezoelectric plasma applied at 10-and-20 mm distances for inactivating *Salmonella* and *Listeria* inoculated on stainless steel surfaces with or without protein residues. The results demonstrated better protection of both bacteria in the presence of the protein soils. This could be due to the protection against the oxidative stress provided by the protein water complex, as well as the probable quenching of the plasma species in the presence of food soils, resulting in a reduction in bactericidal effectiveness [204]. The plasma inactivation efficacy will also depend on the type of microorganism [196]. Laroussi et al. [205] observed a higher disruption of the *E. coli* cell membrane as compared to the *Bacillus subtilis* cell membrane, which remained unaffected. Several investigations have shown that Gram-negative cells are more susceptible to plasma inactivation than Gram-positive cells because their thin cell wall is easily ruptured, resulting in a leakage of the intracellular components [196].

Cold plasma can be used as a dry disinfection treatment. Several studies have documented the decontamination potential of the different cold plasma sources on industrially relevant contact surfaces (Table 7). Aboubakr et al. [206] investigated the effectiveness of the dry and moist exposure of DBD plasma against *Salmonella enterica* inoculated on stainless steel. The addition of water resulted in a reduction of ~6.5 logs after 3 min of treatment. However, the dry conditions caused a lower reduction of 2.5 logs after 10 min of plasma exposure. It was observed that bacteria treated under dry conditions were more resistant to inactivation as compared to wet bacteria. The addition of moisture improves the bactericidal efficiency of the CAP treatment. The dissociation of the water molecules produces hydroxyl radicals (^●^OH), and the subsequent fusion of these highly reactive radicals results in the formation of H_2_O_2_. This increased formation of the reactive oxygen species (ROS) contributes to higher bacterial inactivation [207]. Sen et al. [198] assessed the efficacy of radio frequency plasma treatment using air, O_2_, N_2_, and water vapor (H_2_O, RH 95%) against the inactivation of *E. coli* K12 dried on stainless steel. The *D*-values observed were the lowest when water vapor (12.9 min) was used as the working gas, followed by air (22.8 min), nitrogen (37.1 min), and oxygen (62.8 min). The ionization of water molecules into H^−^ and ^●^OH radicals (Equation (3)) in the plasma chamber usually leads to lower *D*-values and, thus, greater inactivation.

(3) H2O+e−→OH●+H−

Cold plasma offers a broad spectrum of antibacterial activity and is environmentally safe [193]. As a non-thermal technique involving low-to-moderate temperatures, it does not degrade the product characteristics, making it superior to conventional processes. It provides surface treatment and can be effectively applied for the treatment of temperature-sensitive products. Moreover, as opposed to chemical procedures, there is no production of hazardous residues [195,199]. However, the transition from lab scale to industrial scale is challenging due to the equipment size, working gas, RH conditions, and microbial resistance [196].

## 5. Wet Disinfection Methods for Microbial Inactivation in Dry Food Processing Facilities

Adequate sanitation and/or disinfection practices are needed to control the entry of microorganisms into the dry food industry. However, the dry food facilities prefer dry disinfection methods over wet disinfection methods. The greatest difficulty associated with dry disinfection techniques is that they may not eliminate all food soil and surface-attached microorganisms [210]. This necessitates the periodic application of wet disinfection techniques. Cleaning and disinfection operations require the use of toxic-free processes. Common chemical agents used for equipment disinfection include peracetic acid, H_2_O_2_, ozone, chlorine, chlorine dioxide, sodium hypochlorite and quaternary ammonium compounds. However, large amounts of water are needed for surface cleaning in these operations to get rid of any leftover disinfectants. Moreover, bacteria may develop resistance to the overuse of disinfectants due to chromosomal gene mutation [211]. The following are some of the selected conventional and novel wet disinfection methods which can be used in the dry food industry. The antimicrobial mechanisms of the wet disinfection methods are discussed in the following sections in more detail.

### 5.1. Conventional Wet Disinfection Methods

#### 5.1.1. Quaternary Ammonium Compounds

Quaternary ammonium compounds (QACs) are slow acting [212] cationic surfactants extensively used for cleaning and disinfection. They are composed of a positively charged nitrogen center and are surrounded by the aryl or alkyl groups. Hydrophobic cations in QACs bond with acidic phospholipids in microbial cell membranes, thereby preventing the uptake of nutrients and the efflux of waste products [213]. QACs attack the phospholipid bilayer of the membrane in bacteria. Cationic nature helps to invade the bilayer by displacing ions, which causes membrane disorganization [106,214]. Hydrophobic tail interacts with lipids and proteins, resulting in denatured amino acid chains and disrupted lipids.

R-N^+^(CH_3_)_3_ + phospholipid bilayer → membrane disorganization → membrane rupture

QAC + cell membrane → lipid disruption + protein damage → cell lysis

A maximum concentration of 200 mg/L of QAC-based sanitizers is permitted in food processing facilities [215]. QACs have foaming properties, are colorless, odorless, and effective at a broader pH range [212]. They are neither corrosive nor irritant to the skin and possess superior germicidal and wetting properties [216,217].

The antimicrobial efficacy of QACs depends on the microorganism. Al-Qadiri et al. [212] observed that 200 mg/L QUAT reduced *Listeria monocytogenes* and *Staphylococcus aureus* by >5 log, whereas *Salmonella* Typhimurium and *E. coli* inoculated on wooden cutting boards were reduced by <5 log. Gram-positive bacteria, due to the absence of an outer membrane, are vulnerable to QAC-based disinfectants. In another study, Mustpha et al. [218] reported that QAC (50 ppm for 1 min) effectively reduced the number of *Listeria monocytogenes* cells dried for 1 h and 24 h on stainless steel by >4 log.

The disinfection efficacy of QACs will be affected in the presence of organic residues [58]. Li et al. [61] evaluated that benzalkonium chloride (BKC) treatment at 0.5 mg/L for 10 min reduced *S*. *aureus* dried on the stainless steel to below the detection limit. However, a lesser reduction of 0.28 logs was observed in the presence of milk sediments. It was reported that organic layers can block chemicals from entering the cytoplasm of cells.

For surface disinfection, the QACs should be employed at the appropriate concentration [37]. However, increasing the disinfectant concentration may not necessarily have a linear relationship with bacterial inactivation [219] and can promote antibiotic resistance. Benzalkonium chloride (1% for 30 min) applied at 1:1, 1:2, and 1:4 against *E. hirae* inoculated on the stainless steel resulted in a log reduction of 6.30 ± 0.11, 6.31 ± 0.08, and 5.82 ± 0.10 respectively. The chemical properties of the QACs permit their adhesion to the contact surfaces and give a lasting biocidal effect. Ríos-Castillo et al. [219] dried a mixture of benzalkonium chloride 1.0% + NaOH (0.20%) + NaClO (1%) on stainless steel for 1 h and 24 h and evaluated its immediate and long-term antibacterial efficacy against *S. aureus*. Disinfectants dried for 1 and 24 h resulted in a greater reduction of 6.74 ± 0.19 and 6.89 ± 0.13 log, respectively.

Moreover, the germicidal efficacy of QACs will vary depending on the formulations’ pH and concentration of the active ingredient. Kim et al. [220] observed that regardless of the strain type, QAC (pH 12.04) having alkyl dimethyl benzyl ammonium chloride and n-alkyl dimethyl ethylbenzyl ammonium chloride as a microbicide reduced dry *Enterobacter sakazakii* cells on the stainless steel to <1.48 log CFU/coupon after 1 min of treatment. The study postulated the significance of highly alkaline conditions in rupturing the cell membrane and causing cell death. The bactericidal effect of QACs is due to the generation of electrostatic bonds with the cell membrane, resulting in protein denaturation and membrane disruption [38]. They disrupt the peptide bonds of protein moieties, which leads to the instability of the cell membrane and the cytolytic leaking of intracellular components (Figure 5) [221]. The primary drawback of QACs is the formation of bacteriostatic film on food processing equipment, which can lead to residual accumulation. Moreover, they are incompatible with anionic synthetic detergents and have a higher dilution requirement for their germicidal effect [216,217].

#### 5.1.2. Peracetic Acid

**Figure 11:**
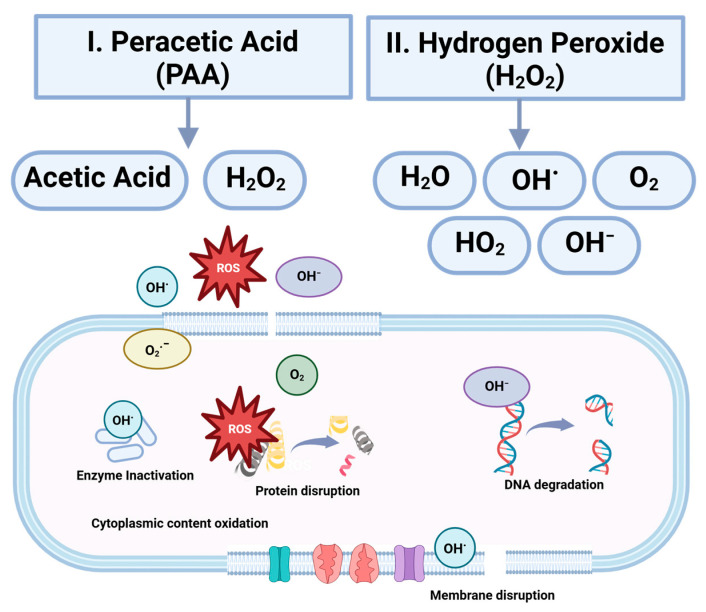
Disinfection mechanism of hydrogen peroxide and peracetic acid. Created with BioRender.com.

In an aqueous solution, peracetic acid (PAA) essentially exists in the chemical equilibrium mixture of acetic acid and H_2_O_2_ [222]. PAA is a potent disinfectant that has a broad spectrum of activity against vegetative bacteria, viruses [223], and spores [222,224]. It functions as a non-rinse eco-friendly disinfectant because its decomposition produces acetic acid, water, and oxygen [39]. The biocidal effect of PAA is due to the oxidation of the cellular macromolecules, resulting in cytoplasmic disruption (Figure 11). The bactericidal action is also linked to the formation of H_2_O_2_ as an intermediate degradation product [225].

CH_3_CO_3_H → CH_3_COOH + H_2_O_2_ (4)

H_2_O_2_ + O_2_ → ⋅OH + O_2_^−^ (5)

PAA generates oxidative stress by producing ROS and free radicals, which react with the microbial cell. Bilayer lipids could be solubilized, and damaged cells would not be able to survive. Dominant reactive species in PAA are believed to be peroxyl radicals (CH_3_COO⋅). This is a highly unstable and reactive species, which is a major contributor to the antimicrobial properties of PAA. Other reactive species generation pathways are as follows [226].

CH_3_COOOH → CH_3_COO⋅ + ⋅OH (6)

These radicals not only react with the cell components but also within themselves and generate more species:

CH_3_COOH + ⋅OH → CH_3_CO⋅ + O_2_^−^ + H_2_O (7)

CH_3_COOH + ⋅OH → CH_3_COOO⋅ + H_2_O (8)

CH_3_COO⋅ → ⋅CH_3_ + CO_2_ (9)

PAA is a weak acid that is very efficient at low concentrations but can also be utilized effectively at alkaline pH when used in higher concentrations. It is thermodynamically unstable, especially in its diluted form, and must be stored at cold temperatures in its original containers [38].

The majority of the research on PAA disinfection has focused on food and biofilms. The FDA has approved the usage of PAA in the wash water for fruits and vegetables at a maximum concentration of 80 ppm [222]. Limited studies have evaluated the effectiveness of PAA against the dry microorganisms adhered to food-contact surfaces (Table 8).

Dried cells have a greater resistance to disinfectants than their planktonic counterparts. The effectiveness of PAA (pH 5.24), both on dry *Enterobacter sakazakii* 3231 on stainless steel and in a planktonic solution, was studied [220]. Planktonic cells were reduced to below the detection limit of 0.30 log CFU/mL after being treated with PAA for 1 min, while dry cells observed a lesser reduction of 1.58 log CFU/coupon. Desiccation triggers the starvation phase in cells, which increases their resilience to various stresses. Moreover, PAA’s ability to disinfect is reduced in the presence of organic residues. In the same experiment, a 1 min PAA treatment reduced the number of planktonic and surface-dried cells in the presence of infant formula by 0.94 log CFU/mL and 0.58 log CFU/coupon, respectively.

Moreover, the PAA disinfection efficacy will vary based on the type of surface, concentration, and treatment time. Choi et al. [223] reported that human coronavirus 229E (HCoV-229E) dried on stainless steel was reduced by 4.73 ± 0.09 to 1.06 ± 0.10 log10 TCID50/coupon when treated with PAA at concentrations between 50 and 200 ppm for 5 min. However, HCoV-229E dried on polypropylene exhibited a reduction in viral titers from 4.70 ± 0.04 to below the detection limit (1.0 log10 TCID50/coupon) following treatment with 50–200 ppm PAA for 5 min.

Spores have a very high resistance to peroxyacetic acid as compared to vegetative bacteria and fungi. Kreske et al. [224] compared the effectiveness of peroxyacetic-based sanitizer (40 and 80 µg/L), chlorine (10 to 100 µg/mL), and chlorine dioxide (10 to 200 µg/mL) on the inactivation of *Bacillus cereus* spores on the stainless steel. The efficacy of the sanitizers was assessed by drying the spores in the presence of water and 5% horse serum. Under all the treatment conditions, a higher susceptibility of the bacillus cereus spores in the order of chlorine > chlorine dioxide > peroxyacetic acid was observed.

#### 5.1.3. Hydrogen Peroxide

Hydrogen peroxide (H_2_O_2_) is a fast-acting, versatile disinfectant with extensive biocidal activity against a wide range of bacteria [232], viruses [233], and spores [234]. It is thermodynamically unstable, and the decomposition of H_2_O_2_-based disinfectants generates water and oxygen, making them environmentally safe. (Equation (10) [230].

(10) $$H_{2}O_{2}+H_{2}O_{2}\to \;2H_{2}O+O_{2}$$

The bactericidal effects of H_2_O_2_ are a direct result of its oxidizing properties, which include the oxidation of biomolecules (proteins, nucleic acids, and lipids), peroxidation of lipid membranes, and inhibition of enzymes, resulting in genomic cell damage (Figure 11) [235]. The principal reactive oxygen species can be produced via two distinct pathways. The reaction of superoxide with the H_2_O_2_ yields a highly reactive hydroxyl radical (^●^OH) [236] (Equation (11)).

(11) O2−●+H2O2→O2+OH−+OH●

In addition, in Fenton’s reaction, transition metal ions decompose H_2_O_2_ to generate a highly reactive hydroxyl radical [236] (Equation (12)).

Fe^2+^ + H_2_O_2_ → Fe^3+^ + OH^−^ + ^●^OH (12)

It possesses better material compatibility and does not produce any toxic byproducts when in contact with organic waste. Conventional procedures such as spraying and immersing entail direct administration of antimicrobial chemicals to the contact surfaces. For the disinfection of food processing plants, fumigation-based strategies are routinely employed. They offer the distinctive advantages of greater dispersion and improved penetrability [237].

H_2_O_2_ decomposes at higher temperatures and is highly corrosive. It can also be used in combination with other agents [235]. H_2_O_2,_ in combination with PAA, is a potent oxidant and environmentally safe [238]. Kim et al. [220] tested the effectiveness of a disinfectant based on peroxyacetic acid (5.1%) and hydrogen peroxide (21.7%) against *E. sakazakaii* which had been dried on stainless steel in water and infant formula. The *E. sakazakaii* cell counts were reduced to 1.48 log CFU/coupon after 10 min of treatment with the disinfectant in both drying carriers (water and infant formula). In another study by Gulati et al. [238], a peroxyacetic acid (15%) + H_2_O_2_ (11%)-based sanitizer at a 1:2000, 1:1000, and 1:500 dilution reduced Feline calicivirus (FCV) dried on the stainless steel by 0.4 ± 0.1, 0.6 ± 0.05, and 3.00 ± 0.0 log10 FCV, respectively.

The fundamental issue with foam- or gel-based disinfectants is their limited accessibility in the niche areas of food processing equipment and machines [237]. Therefore, the distribution of the disinfectant in the form of an aerosol and mist is a possible alternative for surface disinfection [237]. A H_2_O_2_-based mist system (5–10%) is commercially used in the healthcare setting [239]. Choi et al. [240] examined the survival rates of *E. coli* O157:H7, *Listeria monocytogenes*, and *Salmonella* Typhimurium after being air-dried for 2 h on stainless steel and then equilibrated for 7 days at 70% RH. All three pathogens were below the detection limit (1 log CFU/mL) following a 60 min treatment with a 0.25% H_2_O_2_ aerosol.

Møretrø et al. [232] investigated the efficacy of a 5% H_2_O_2_ mist containing 0.005% silver for whole-room disinfection under conditions relevant to the food industry. The suspension of *Listeria monocytogenes* was inoculated onto the stainless steel without drying, and another set of coupons was dried for one hour in the biosafety cabinet. H_2_O_2_ was applied to both sets of coupons. Under all tested conditions of H_2_O_2_ concentration (40–80 ppm), contact time (53–126 min), and temperature (20 °C), the cells in suspension exhibited a decrease of at least 5 logs. A greater reduction may be attributable to the dissolution of H_2_O_2_ in liquid-phase suspension cells. However, the dried *Listeria monocytogenes* cells were more resistant and showed an overall reduction of 1.5 log. The effectiveness of H_2_O_2_ against surface-dried microorganisms has only been studied to a small extent (Table 9). Decontamination efficacy for H_2_O_2_ in mist/aerosol has been established against several molds dried on food-contact surfaces. Kure et al. [237] discovered that exposure to H_2_O_2_ mist (40–80 ppm) for 2 h resulted in a >3-log reduction in *Alternaria alternata* adhered to stainless steel. The primary drawback is that H_2_O_2_ is highly sensitive to the presence of heavy metals. In addition, for a better antimicrobial effect, a concentration ≥3% is necessary [102].

#### 5.1.4. Sodium Hypochlorite

Sodium hypochlorite, generally known as household bleach, is a major disinfectant used in the food industry [243]. NaOCl is relatively inexpensive and easy to use. It has biocidal potential against bacteria [89], viruses [238], and spores [244]. NaOCl’s active form, ^−^OCl, has germicidal properties and can potentially damage DNA and limit protein synthesis (Figure 6) [243]. Sodium hypochlorite in an aqueous solution is in equilibrium with ^−^OCl, HOCl, and Cl_2_ (Equations (14) and (15)).

NaOCl + H_2_O → HOCl + Na^+^ + OH^−^ (13)

HOCl ⇌ ^−^OCl + H^+^ (14)

HOCl + H^+^ + Cl^−^ ⇌ Cl_2_ (aq.) + H_2_O (15)

Bridges et al. [245] studied the internal oxidative stress response in *E-coli* 0157:H7 induced by NaOCl and ClO_2_. The researchers reported that selected genes for stress response were significantly increased by NaOCl, whereas ClO_2_ did not show a significant difference. Possibilities for significant damage to the lipid bilayer and other areas vulnerable to oxidative damage arise from increased vulnerability to oxidative stress [245,246].

Sodium hypochlorite solutions with an acidic pH between 4 and 6 have a greater biocidal action due to the presence of a greater amount of hypochlorous acid [247]. As the pH of the solution drops below 4, HOCl decomposes into Cl_2_ (Equation (15)). The appropriate NaOCl concentration is determined by the total available chlorine, which includes both free and combined chlorine [247]. As a no-rinse food-contact surface sanitizer, the maximum concentration of sodium hypochlorite permitted by the FDA is 200 ppm of available chlorine [248]. Table 10 provides a summary of the studies demonstrating the effectiveness of NaOCl in inactivating dry microorganisms from food-contact surfaces.

The efficacy of the NaOCl treatment varies depending on concentration, sanitizer exposure time [218], and the presence of organic residues. Takahashi et al. [54] observed complete inactivation of murine norovirus after 30 min of drying on stainless steel after treatment with NaOCl at 1000 ppm for 5 min. However, when murine norovirus (MNV-1) was dried on stainless steel in the presence of organic residues (cabbage, ground pork, and lettuce), its survival and resistance to the disinfectant increased. The efficiency of NaOCl is also dependent on the drying time of microorganisms on the contact surface [254]. Mustpha et al. [218] reported that *Listeria monocytogenes* was more susceptible to NaOCl after being dried on stainless steel for 24 h compared to 1 h. This could be attributed to the variations in drying time and humidity.

A NaOCl treatment (2700 ppm for 1 min) of MNV-1 on stainless steel resulted in a greater reduction under wet conditions (6.8 log_10_ reduction) than under dry conditions (5.9 log reduction) [254]. Furthermore, the resistance of the foodborne pathogens to disinfectants will vary based on the growth phase, such as the log, stationary, and long-term stationary (LTS) phases. Djebbi-Simmons et al. [89] compared the resistance of the low-, medium-, and high-microbial-load cells dried for 24 h on the stainless steel against disinfection by sodium chlorite (0.0095%). Under the tested condition, cells in the LTS phase were more resistant to disinfection than those in the log and stationary phases. Bacteria, upon entry to the stationary phase, initiate the expression of the alternative sigma factor to prevent desiccation tolerance. The requirement for handling precautions is one of sodium hypochlorite’s primary drawbacks. In addition, antimicrobial resistance can be avoided through the rotation of disinfectants [255].

### 5.2. Novel Wet Disinfection Methods

#### 5.2.1. Electrolyzed Water (EW)

**Figure 12:**
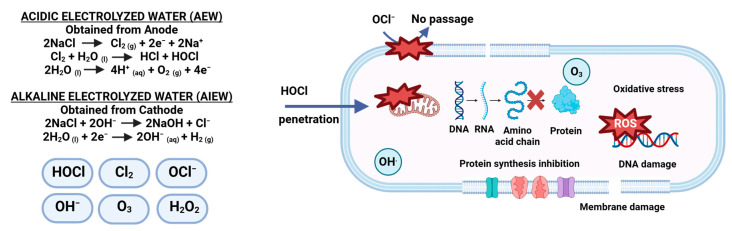
Disinfection mechanism of electrolyzed water. Created with BioRender.com.

Electrolyzed water (EW) or electrochemically activated water (ECA) has a broad spectrum of antimicrobial activity against bacteria [256], fungi, viruses, and spores [257]. It is produced by electrolyzing a diluted NaCl solution [256] and produces active chlorine concentration (ACC) compounds (Cl_2_, OCl^−^, and HOCl) as well as reactive oxygen species (H_2_O_2_ and O_3_) (Figure 12).

H_2_O → •OH + H^+^ + e^−^ (16)

O_2_ + e^−^ → O_2_^−^ (superoxide) (17)

The presence of the oxidizing compounds increases the oxidation–reduction potential (ORP) of EW. Liao et al. [258] examined the role of ORP in inactivating *E. coli* suspensions and observed that as ORP increased, the outer and inner membranes of *E. coli* were more severely damaged. According to several studies, sulfhydryl groups on cell surfaces are oxidized in the presence of a greater ORP [259]. Multiple factors, including pH [260], ACC, ORP, surface type [250], and the presence of organic matter, govern EW’s microbial efficacy.

Hypochlorous acid (HOCl) with a pH of 5.0–6.5 is the active chlorine form present in EW [260]. HOCl is electrically neutral and has a low molecular weight that is equivalent to that of H_2_O. The germicidal action of HOCl results from its passive diffusion into the cell wall and membrane, and the production of the ^●^OH radicals causes the oxidation of cellular components [256]. HOCl is a relatively weak acid with an approximate pKa of 7.46 [256]. At a pH < 4.0, it dissociates to generate Cl_2_, while at a higher pH, the reversible reaction causes HOCl to decompose into H^+^ and OCl^−^. Ionized OCl^−^ possesses low bactericidal action. It is unable to penetrate the hydrophobic lipid bilayer and thus only oxidizes the outer cell wall components. Moreover, the reactive oxygen species (H_2_O_2_ and ozone) produced by EW contribute to its antimicrobial activity [259]. The biological pathway of reaction would be as follows [261]:

(18) $$\mathrm{HOCl}+R\text{-}\mathrm{SH}\;(\mathrm{membrane}\;\mathrm{protein})\;\to \;R\text{-}\mathrm{SOH}\;(\mathrm{oxidized}\;\mathrm{protein})+{\mathrm{Cl}}^{-}$$

This reaction with sulfhydryl groups in proteins results in the denaturation of membrane proteins and the leakage of intracellular contents. HOCl acts by oxidatively damaging microbial DNA and essential proteins, which inhibits cell replication and function. HOCl can cause strand breaks in DNA and the oxidation of key enzymes, leading to cell death.


(19) HOCl+DNAbases (Adenine,Guanine) → oxidizedbases(8-oxoguanine)

(20) HOCl+R-SH(Cysteine) → R-SOH → R-S-S-R(disulfide bond formation)

EW can be divided into acidic electrolyzed water (AEW), neutral electrolyzed water (NEW), and alkali electrolyzed water (AIEW) based on the various electrolytes, equipment, and electrolysis conditions [256]. AEW, with a pH of 2.3–3.7, ORP > 1000 mV, and ACC 10–100 ppm, is a natural sanitizer [250]. Han et al. [262] reported that AEW generated with 3 g/L NaCl reduced the *Escherichia coli* biofilm cell population by more than 67%, whereas the cell populations in the biofilm formed by *Vibrio parahaemolyticus* and *L. monocytogenes* were reduced to 52 and 82%, respectively. Ni et al. [263] reported that exposure to AEW decreased the dehydrogenase activities of *E. coli* and *S. aureus*, resulting in the rapid loss of intracellular DNA, potassium, and proteins and an increase in the permeability of the bacterial membrane. However, the application of AEW is limited by various factors, such as equipment corrosion and skin irritation. In addition, AEW can lose its bactericidal activity during storage, and a low pH can favor the loss of Cl_2_ gas.

Neutral, acidic water (NEW) (pH 7.0–8.0; ORP 750–900 mV) is more effective in disinfecting than acid-electrolyzed water (AEW) [250]. It has been eported that NEW is non-corrosive and does not lose its antibacterial properties upon storage. It can be substituted for sodium hypochlorite (NaOCl), a chemical sanitizer widely used for surface disinfection [264]. Deza et al. [250] compared the efficacy of NEW (pH: 7.76 ± 0.35; ACC: 64.11 ± 6.29 mg/L) and NaOCl (8.11 ± 0.41; ACC: 62.3 mg/L) in disinfecting plastic cutting boards inoculated with *Listeria monocytogenes*. There was no significant (*p* ≥ 0.05) difference in the reduction efficacy of NEW and NaOCl after treatment for 1 min. A similar study by Deza et al. [250] observed no significant difference in the effectiveness of NEW (pH 8.0 ± 0.5; ACC: 60 mg/L) and NaOCl (ACC: 60 mg/L) in reducing *E. coli* and *Listeria monocytogenes* inoculated on stainless steel and glass surfaces after 1 min of treatment.

EW is a novel disinfectant with numerous advantages. It does not involve the use of harmful chemicals and has lesser environmental impacts than conventional chemical sanitizers. It is cost-effective, and it does not affect the sensory properties of the foods. EW is more convenient for on-site manufacturing [259,265], as the primary expenses are the initial outlay for the EW generator, chemical salts, and water.

The loss of EW’s antibacterial characteristics due to a lack of Cl_2_, H^+^, and HOCl via electrolysis and the high initial cost of the necessary equipment are the biggest drawbacks. Furthermore, the powerful chlorine gas released by different EW generators when the pH is below 5 may cause operator discomfort. Antimicrobial action in EW can also be diminished due to improper storage. Moreover, the presence of organic matter neutralizes the disinfection efficacy of EW and reduces its shelf life; thereby, an additional cleaning step is necessary before its application [259]. Diverse studies have advocated combining EW with other disinfection technologies to overcome these shortcomings.

#### 5.2.2. Plasma Activated Water

The plasma treatment of water results in the generation of plasma-activated water (PAW) [194]. It is a non-thermal technique with broad antimicrobial activity against bacteria [266], viruses [267], and spores [268] and can also be effectively used for surface decontamination [269,270]. PAW can be generated either at the interface between gas and liquid or in the liquid phase, due to the dissolution of gas bubbles containing the plasma reactive species [270]. RONS production will vary depending on whether the discharge is submerged, on the water’s surface, or in bubbles. It is an easy-to-use technology and produces no hazardous byproducts, unlike chemical disinfectants [271]. The distinctive advantage of PAW compared to cold plasma is that it can be produced and stored and later utilized for surface disinfection, or it can be produced continuously and circulated [269]. However, certain ROS have a shorter shelf life, thereby necessitating improvement of the procedure. Low-temperature storage provides plasma radicals with higher stability [64].

Plasma discharge dissociates water molecules and generates short-lived OH radicals and electrons. The subsequent reactions between OH and electrons generate a sequence of chemical reactions [272].

^●^OH + ^●^OH → H_2_O_2_ (21)

H_2_O + e^−^ → ^●^OH + ^●^H + e^−^ (22)

H_2_O + e^−^ → ^●^OH + H^+^ + 2e^−^ (23)

H + O_2_ → HO_2_ (24)

O_2_ + O^●^ → O_3_ (25)

NO_2_^−^ + H^+^ → HNO_2_ (26)

NO_2_^●^ + ^●^OH → HNO_3_ (27)

The antimicrobial activity of PAW results from the production of reactive oxygen species, reactive nitrogen species, charged particles, ions, and electrons. Moreover, the low pH and the high oxidation–reduction potential (ORP) also contribute to the antimicrobial effect of PAW [272]. The effectiveness of PAW can be increased by optimizing the process parameters, such as the applied voltage, gas type, flow rate, reactive species composition, presence of organic matter, and exposure period. Moreover, the microbial properties, such as species type and state (planktonic, dry, or biofilm), will also influence the efficacy [273,274].

**Figure 13:**
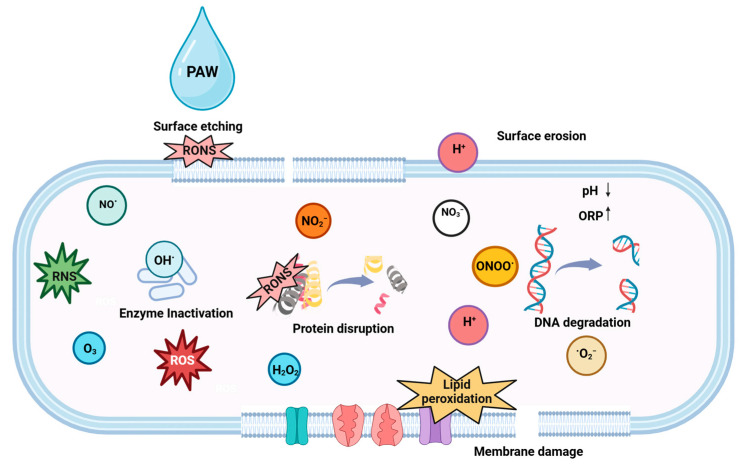
Disinfection mechanism of plasma activated water (PAW). Created with BioRender.com.

The antimicrobial effect of PAW is attributed to the electroporation-induced surface etching and membrane permeability caused by the production of charged particles. In addition, the generated electric field increases membrane permeability, and the interaction between biological molecules causes DNA damage, protein disruption, and lipid peroxidation, which ultimately results in cell death (Figure 13). ROS production across the membrane induces oxidative stress and the oxidation of polyunsaturated fatty acids, leading to lipid peroxidation. In addition, as membrane permeability increases, water can enter the cell and cause swelling [275]. Baek et al. [276] dried *S. aureus* ATCC 27213 for 60 min on stainless steel and treated it with PAW generated using an atmospheric-pressure plasma dielectric generator (2.2 kHz and 4.2 kV for 10 min) and observed a 1.07-log reduction in cell counts. Most of the antimicrobial research has focused on the use of different generations of plasma-activated water against the treatment of biofilms. However, limited research has examined its effectiveness against surface-dried bacteria on contact surfaces.

## 6. Commercial Status and Future Perspectives

Recent incidents involving the persistence of microorganisms on food-contact surfaces and their subsequent contamination of food products have sparked significant concern. The current dry-cleaning process in industries involves a step-by-step approach, beginning with preparing cleaning materials and emptying all products. Some devices are disassembled, and removable parts are safely stored. Each piece of equipment has a separate cleaning protocol that utilizes brushes, sweeping, and vacuuming. If allergens or toxic materials are present, air blowing is avoided. Certain surfaces require scraping without damaging them and vacuuming them to remove residues. Later, rags or clothes are used to eliminate grease and lubricant near seals. Following this, trial runs are conducted along with inspections. After the final inspection and sanitization, production begins anew. According to the production process, a suitable method is chosen for dry cleaning. Some common techniques are vacuuming, using compressed air, dry ice blasting, brushing and scraping, wiping, and dry steam cleaning. Strict hygiene and disinfection practices should be followed to limit the attachment of microorganisms to food-contact surfaces. The attachment of microbial pathogens inevitably occurs and consequently increases the risk of foodborne illnesses. Before sanitation and or disinfection, thorough cleaning must be performed. Table 11 summarizes the advantages and disadvantages of the dry and wet disinfection methods in detail. Dry disinfection is applied for hygroscopic goods, as exposure to water might form hard deposits. Moreover, the introduction of moisture can potentially promote microbial growth and lead to subsequent contamination [37]. While the USFDA has approved UV radiation for food product decontamination, there are no such guidelines for food-contact surface disinfection applications. It is recommended for use based on their specifications and good manufacturing practices [277]. Moreover, disinfecting with UV lights has its limitations since it may not reach all of the processing and inaccessible parts of the dry food facility [181]. Superheated steam, also known as dry steam, is another promising dry technology that has recently been investigated for use in the surface disinfection of the low-*a_w_* food processing industry [278]. Fumigation with gases can be an effective dry cleaning method because of its rapid action, on-site production, and great diffusivity compared to alternative methods [237]. Ozone sanitation is commercialized and extensively utilized [e.g., Absolute Ozone^®^ (Edmonton, AB, Canada) (https://absoluteozone.com/ accessed on 19 February 2025), Ozone Solutions (San Antonio, TX, USA) (https://ozonesolutions.com/ accessed on 19 February 2025), and Spartan Environmental Technologies (Mentor, OH, USA) (https://spartanwatertreatment.com/ accessed on 19 February 2025)]. Ozone has GRAS status and is approved for direct food contact by the USFDA [169]. Chlorine dioxide is another commercially available method used for disinfecting food surfaces [e.g., PureLine (Bensenville, IL, USA) (https://www.pureline.com/ accessed on 19 February 2025), ClorDiSys Dolutions, Inc. (Somerville, NJ, USA) (https://www.clordisys.com/ accessed on 19 February 2025), and Industrial Fumigant Company (Lenexa, KS, USA) (https://indfumco.com/ accessed on 19 February 2025)].

Wet cleaning with chemical antimicrobials is effective in eradicating pathogenic microorganisms, but it also introduces water that can harbor microorganisms. Chemical sanitizing and disinfecting agents such as PAA, H_2_O_2_, quaternary ammonium compounds, and sodium hypochlorite are used commercially for food-contact surface disinfection according to the USFDA regulations (Code of Federal Regulations Title 21 Section 21CFR178.1010).

Electrolyzed water is an evolving and commercially available sustainable option that can be used for food surface disinfection [e.g., EAU Technologies (Kennesaw, GA, USA) (https://www.eautechnologies.com/ accessed on 2 March 2025)]. Moreover, electrolyzed water has applications in agriculture, pharmaceuticals, the hospitality industry, and restaurants (e.g., Envirolyte Industries International Ltd. [Tallinn, Estonia, Europe) (https://www.envirolyte.com/ accessed on 2 March 2025) and EWCO (Miami, FL, USA) (Electrolyzed Water Co.) (https://ewco.com/ accessed on 2 March 2025)] promote EW’s application in wastewater disinfection and the disinfection of food-contact surfaces). Cold plasma and plasma-activated water are additional novel dry and wet disinfection technologies with potential applications in the dry food industry [e.g., STERAMIST (Frederick, MD, USA (https://steramist.com/ accessed on 2 March 2025) and Relyon plasma (Regenburg, Germany) (https://www.relyon-plasma.com/?lang=en accessed on 2 March 2025)] have commercialized cold plasma for surface sanitization in the food and medical industries, respectively, while Vital Fluid (Eindhoven, Netherlands) (https://vitalfluid.com/ accessed on 2 March 2025) and Plasma Waters (Miami, FL, USA) (https://plasmawaters.com/ accessed on 2 March 2025) have developed small-scale equipment and procedures for creating plasma-activated water in the laboratory). Surface decontamination using plasma-activated water is an emerging method.

The complete removal of pathogens from contact surfaces is challenging. Therefore, strict operating hygienic practices coupled with disinfection techniques should be followed to ensure food safety control and to avoid potential recontamination in the dry food industry. Extensive research is required to comprehend the long-term survival of dried pathogenic microorganisms on food-contact surfaces and to investigate the inactivation behavior using various novel and conventional disinfection techniques. Moreover, a greater comprehension of the survival and growth mechanisms of microorganisms will aid in the development of the cleaning and disinfection processes.

## 7. Concluding Remarks

The long-term persistence and survival of pathogens under the dry conditions of low-*a_w_* food processing facilities has been the subject of substantial study. Controlling biological hazards in dry food processing facilities is an ongoing challenge. Disinfection procedures should be used with consideration of the types of foods being manufactured, the frequency of cleaning, and the presence of organic residues on food-contact surfaces. To reduce the likelihood of bacterial persistence and subsequent cross-contamination, it is important to select a disinfection approach that is both easy to implement and cost-effective. Generally, wet disinfection methods are used in food industries; however, due to the chance of microbial growth, dry disinfection methods are preferred for dry food processing facilities. The dry and wet disinfection methods include dry heat, UV light, alcohol-based methods, gaseous ozone, ClO_2_, PAA, H_2_O_2_, quaternary ammonium compounds, and electrolyzed water. There is a risk of chemical residue being left on processing equipment, and the misuse of chemical disinfectants can lead to the development of resistant strains. Moreover, the addition of moisture can promote the formation of caked-on organic residues, resulting in the entrapment and subsequent growth and proliferation of pathogenic microorganisms, thereby posing a risk to food safety. This highlights the need for research into alternative, less costly, and more environmentally friendly methods of disinfection. While traditional methods provide reliable options, their limitations necessitate the adoption of innovative approaches. By addressing the challenges of scalability, cost, and public perception, novel methods can play a crucial role in the industry’s future. Some future directions are going to be focused on combined treatments, automation and standardization, developing improved training programs, and addressing consumer education to increase the acceptance of novel disinfection technologies. However, the efficacy of cleaning and disinfection procedures, particularly against dry microorganisms, has not been investigated thoroughly. In addition, studies on the genetic mechanism of pathogenic microorganisms in various disinfection procedures are limited. The frequent occurrence of persistent and resistant strains in dry conditions is also a cause for concern. Thus, effective disinfection technologies must be selected to address the aforementioned problems.

## Figures and Tables

**Table 1:** Summary of the studies demonstrating the outbreaks linked with the dry food production environments.

Pathogens	Year	Remarks	References
*Salmonella* Newbrunswick	1965–1966	Inadequate hygiene standards in the spray dryer resulted in the isolation of *Salmonella* from the air filter	[16]
*Salmonella* Eastbourne	1975	Dust-induced airborne contamination of chocolate	[17]
*Salmonella* Agona	1998 and in 2008	Long-term persistence of *Salmonella* in the dry environments of the cereal manufacturing plant	[18]
*Salmonella* Wandsworth and *Salmonella* Typhimurium	2007	The initial examination revealed that the puffed rice snack was contaminated with *Salmonella*. The recall was expanded to include other items containing the same components or processed with the same equipment	[19]
*Salmonella* Schwarzengrund	2007	Two prominent brands of dry dog food related to *Salmonella* contamination manufactured in the same facility	[20]
*Salmonella* Tennessee	2007	Outbreak related to peanut butter of 2 different brands (Peter Pan and Great Value brand) manufactured in the same facility	[21]
*Salmonella* serotypes Montevideo, Newport, and Senftenberg	2009	Contamination of pistachio nuts and pistachio-nut-containing products produced in the same facility	[22]
*Salmonella*	2011	Presence of *Salmonella* in the air, broom, floor, and processing equipment of the feed mills	[23]
*E. coli* O157:H7	2011	*E. coli* contamination of hazelnuts and hazelnut-containing products, sourced from the same distributor	[24]
*Salmonella* Bredeney	2012	Outbreak related to peanut butter of 2 different brands manufactured in the same facility	[25]
*Salmonella* Montevideo and Salmonella Mbandaka	2013	*Salmonella* infection of tahini sesame paste. To avoid the potential risk of Salmonella, subsequent batches produced on the same production line were also recalled	[26]
*Salmonella Braenderup*	2014	Contamination of almond and peanut butter manufactured in the same plant	[27]
*Salmonella* Montevideo and *Salmonella* Senftenberg	2016	Pistachios contaminated by the same farms’ production	[28]
*Salmonella* Paratyphi B	2016	Nut butter, sprouted nut butter, and all other items made on the manufacturing line were recalled owing to potential contamination	[29]
*E. coli*	2016	Recalled various varieties of flour manufactured at the same plant	[30]
*Salmonella* Typhimurium	2018	Multiple products of dried coconut contamination	[7]
*Salmonella* Newport	2018	The outbreak was linked to two distinct brands of dry shredded coconut manufactured in the same plant	[31]

**Table 2:** Summary of studies demonstrating the desiccation survival of microorganisms with or without the presence of food residues.

Pathogens	Contact Surface	Drying Conditions	Food Sediment	Log Reduction	Reference
*L. monocytogenes* N53-1	Stainless steel	Storage at 15 °C at 43% RH for 91 days	Smoked salmon juice with 5% salt	~4.5	[49]
*S. enterica*	Stainless steel	Storage at 6.5 °C at 60–70% RH for 168 h	Chard (66.8 g/100 mL)	6.26	[50]
			Romaine lettuce (66.8 g/100 mL)	7.68	
*L. monocytogenes*	Stainless steel	Air-dried, storage for 30 days at 25 °C	Minced tuna (100 g/100 mL)	~5	[51]
			No food residue	>7	
*L. monocytogenes*	Stainless steel	Biosafety drying for 120 h	Soy milk (50%)	0.48	[52]
			No food residue	3.08	
*S.* Enteritidis	Stainless steel	Biosafety drying for 120 h	Soy milk (50%)	1.83	
			No food residue	4.4	
*S. aureus*	Stainless steel	Biosafety drying for 120 min	Carrot juice (50%)	<1	[53]
			Distilled water (no residue)	~2	
Murine norovirus-1 (MNV-1)	Stainless steel	Storage for 30 days	Cabbage (100 g/100 mL)	1.4	[54]
			No food residues	6.2	
*Enterobacter sakazakii*	Stainless steel	Biosafety drying for 2 h, and storage at 43% RH at 4 °C for 60 days	Infant formula	1.07–1.21	[55]
			No food residues	1.73–2.02	
*L. monocytogenes*	Stainless steel	Drying at 43% RH at 15 °C for 23 days	0.5% NaCl	2.46	[47]
			5% NaCl	0.88	
*Salmonella* spp.	Paper discs	Drying for 25 h at 35 °C	No food residues	2.43–3.51	[56]
		Storage of the dried cells at 4 °C for 22–24 months		<1	
*S.* Typhimurium “DS”	Stainless steel	Drying for 80 min at 30 °C, storage at 33% RH at 25 °C for 30 days	No food residues	4.3	[57]
*S.* Typhimurium DT104				1.3	

**Table 3:** Summary of studies demonstrating the log reduction in microorganisms in the presence of food residues and when treated with disinfectants.

Pathogens	Contact Surface	Drying Conditions	Disinfection Technique	Treatment Conditions	Food Sediment	Log Reduction	Reference
*E. coli* O26	Stainless steel	Biosafety drying for 90 min	Benzalkonium chloride	2 mg/L, 10 min	Milk	0.39	[58]
*S.* Typhimurium	Stainless steel	Biosafety drying for 120 min	Sodium hypochlorous acid	0.01% *w*/*v*, 10 min	Carrot	<1	[59]
*S.* Typhimurium	Glass	Biosafety drying for 180 min	Benzalkonium chloride	2 mg/mL, 10 min	Whole egg solutions	<1	[60]
*S. aureus*	Polystyrene	Biosafety drying for 90 min	Benzalkonium chloride	0.5 mg/mL, 10 min	Bovine serum albumin (BSA)	N. D.	[61]
*S. aureus*	Polystyrene	Biosafety drying for 90 min	Benzalkonium chloride	2.0 mg/mL, 10 min	Milk	1.85	[61]
*E. coli*	Stainless steel	Biosafety drying for 120 h	Benzalkonium chloride	500 mg/L, 10 min	Soy milk (25%)	1.5	[52]
*S.* Typhimurium	Glass	Biosafety drying for 180 min	UV-C (254 nm)	1 min	Egg yolk (15%)	~3	[62]

**Table 4:** Surface characteristics of various food-contact materials.

Materials	Contact Angle	Surface Energy Parameters			References
	θ_w_ (°)	γ^LW^ (mJ/m^2^)	γ^+^ (mJ/m^2^)	γ^−^ (mJ/m^2^)	
Stainless steel (type 304, P80 finish)	51.8 ± 9.8	ND	ND	ND	[62]
Stainless steel (type 304, diamond-polished)	76.1 ± 10.6	ND	ND	ND	[62]
Stainless steel (type 304, electropolished)	58.9 ± 4.4	ND	ND	ND	[62]
Stainless steel (type 304, #4 finish)	32.0 ± 3.6	37.9	0.5	1.8	[63]
Stainless steel 304	65.8	39.62	0.0	18.43	[64]
Stainless steel 316 L	48.8	39.0	0.02	36.39	[64]
Stainless steel (type 304)	86 ± 2	35.5	0.0	3.8	[65]
Titanium	42.0	41.32	0.04	41.14	[64]
Glass	73.5 ± 3.1	29.6	0.0	20	[66]
Glass with metal oxide finish (TiO_2_)	59 ± 2	ND	ND	ND	[67]
Glass with metal oxide finish (Fe_2_O_3_)	68 ± 5	ND	ND	ND	[67]
Glass	12 ± 3	39.9	1.5	51.8	[68]
Silicone	122 ± 1.8	12.4	0.0	0.9	[66]
Polyethylene	102 ± 2.4	36.4	0.0	0.6	[66]
Polypropylene	107 ± 3	28.4	0.0	1.7	[66]
Polyurethane	80.4	36.34	0.00	7.85	[64]
Polyvinyl chloride	95.4 ± 2.9	33.9	0.0	5.8	[66]

θ_w_ (°) indicates the contact angle measurement with water. γ^LW^, γ^+^, and γ^−^ indicate the Lifshitz–van der Waals, electron accepting, and electron donating surface energy parameters, respectively. ND indicates not determined.

**Table 5:** Summary of studies demonstrating the use of ClO_2_ in inactivating microorganisms dried on food-contact surfaces.

Pathogens	Contact Surface	Drying Conditions	ClO_2_ Gas Parameters	Log Reduction	References
*S.* Typhimurium	Stainless steel	Biosafety drying for 1 h	20 ppmv, at 15 °C for 30 min	<1	[131]
			20 ppmv, at 25 °C for 30 min	1.5–2.0	
*L. monocytogenes*	Stainless steel	Biosafety drying for 2 h	2 mg/L for 10 min	3.8	[136]
*E. coli* O157:H7	Polyvinyl chloride	Biosafety drying for 1 h	20 ppmv for 15 min	3.0	[131]
*Bacillus subtilis*	Glass	Biosafety drying for 12 h	0.080% for 3 h	>6.5	[137]
	Stainless steel			<5	
*Bacillus thuringiensis*	Wood	Biosafety drying for 3 h	5 mg/L under 85–92% RH for 12 h	3.6	[139]

**Table 6:** Summary of studies demonstrating the use of UV light in inactivating the microorganisms dried on food-contact surfaces.

Pathogens	Contact Surface	Drying Conditions	UV Exposure Conditions	Log Reduction	References
*S. enterica*	Stainless steel	Biosafety drying for 90 min	UV-C light (254 nm) at 656 µW/cm^2^ for 5 s (3.3 mJ/cm^2^)	2.75	[181]
	High-density polyethylene			2.93	
	Waxed cardboard			1.39	
	Polyvinyl chloride			1.91	
*S. enterica*	Stainless steel 304 hairline	Biosafety drying for 4 h	UV-C (254 nm) at 15 W for 0–180 s	>4	[189]
*S. Typhimurium*	Stainless steel	Air-drying for 30 min	UV-C (254 nm) at 250 µW/cm^2^ for 3 min	4.35	[190]
*E. coli* O157:H7				5.2	
*Salmonella* spp.	Electroplated stainless steel	Biosafety drying for 30 min	UV-C (254 nm) at a dose of 0.20 J/cm^2^	3.34	[182]
*L. monocytogenes*				2.89	
*S. aureus*				2.58	
*L. monocytogenes*	Polyurethane	Biosafety drying for 30 min	UV light (254 nm) at 5.53 mW/cm^2^ for 3 s	4.97	[191]
*S.* Typhimurium DT104	Stainless steel	Biosafety drying for 30 min	UV (253.7 nm) at 0.236 ± 0.013 mW/cm^2^ for 30 min	0.82	[192]
	Polypropylene			1.62	

**Table 7:** Summary of studies demonstrating the use of cold plasma in inactivating microorganisms dried on food-contact surfaces.

Pathogens	Contact Surface	Drying Conditions	Plasma Type	Plasma Exposure Conditions	Log Reduction	References
*E. coli*	Stainless steel	Biosafety drying for 30 min	Surface micro-discharge plasma	Air (90% rH, 5 SLM), for 20 min	4.13	[204]
*S. aureus*					3.38	
*S. enterica*	Stainless steel	Biosafety drying for 4 h	Atmospheric pressure plasma jet system	Air (5 SLM), for 14 s	≥6	[189]
*E. coli*	Stainless steel	-	Atmospheric pressure plasma jet system	Air (12 SLM) for 90 s	3.40	[208]
	Polypropylene				3.40	
*S.* Typhimurium	Stainless steel	Biosafety drying for 1 h	Piezoelectric cold atmospheric plasma	15 V, 50 kHz, Air for 300 s at 10 mm distance	3.5	[201]
*S. enterica*	Glass	Biosafety drying for 1 h	Surface dielectricbarrier discharge	7 kV, 13.5 V, Air, 1 cm distance, 4 min	3.0	[202]
*S. epidermidis*	Stainless steel	1–2 h drying at 35 °C	Gliding arc discharge	Nitrogen (0.5 m^3^/h) for 5 min	3.94	[197]
*E. coli*					3.65	
*E. coli*	Wood chopping board	Biosafety drying for 30 min	Atmospheric dielectric barrier discharge plasma	Nitrogen (1.5 lpm) for 60 min	1.6	[209]

**Table 8:** Summary of studies demonstrating the use of PAA in inactivating the microorganisms dried on food-contact surfaces.

Pathogens	Contact Surface	Drying Conditions	PAA Concentration	Log Reduction	References
*A. brasiliensis*	Aluminium	Biosafety drying for 1 h	1000 mg/L at 40 °C	~6	[227]
*Geobacillus stearothermophilus* spores	Stainless steel	Biosafety drying	200 ppm for 5 min	<1.5	[228]
Murine norovirus	Stainless steel	Drying for 18–24 h, soiled with bovine serum albumin	200 ppm for 3 min	N. C. (no cytopathic effect)	[229]
Feline calicivirus	Stainless steel	Biosafety drying for 30 min	15% PAA and 11% H_2_O_2_ at 1:500 dilution	3.00	[230]
Hepatitis A virus	Stainless steel	Biosafety drying for 1 h	200 ppm for 10 min	4.43	[231]

**Table 9:** Summary of studies demonstrating the use of H_2_O_2_ in inactivating microorganisms dried on food-contact surfaces.

Pathogens	Contact Surface	Drying Conditions	H_2_O_2_ Concentration	Log Reduction	References
Feline calicivirus	Stainless steel	Air-drying for 45 min in the biosafety cabinet	H_2_O_2_ (7.5%) for 5 min	4.3	[140]
*E. coli*	Glass	Biosafety drying for 30 min	H_2_O_2_ (5%) micro aerosol mist for 30 min	5.31	[241]
*S.* Typhimurium	Glass	Biosafety drying for 22 h at 25 °C and 40% RH	H_2_O_2_ (2%) for 5 min	4.3	[3]
*S.* Enteritidis	Stainless steel	1 h of biosafety drying of the stationary-phase cells	H_2_O_2_ (3.4%) for 10 min	5.26	[242]

**Table 10:** Summary of studies demonstrating the use of NaOCl in inactivating microorganisms dried on food-contact surfaces.

Pathogens	Contact Surface	Drying Conditions	NaOCl Concentration	Log Reduction	References
*S.* Typhimurium	Plastic cutting board	Biosafety drying for 24 h (using high microbial load)	0.0095% for 2 min	1.75	[89]
*S.* Enteritidis	Glass	Biosafety drying for 22 h at 25 °C and 40% RH	100 ppm for 5 min	5.8	[249]
*L. monocytogenes*	Stainless steel	Biosafety drying for 24 h	200 ppm chlorine for 5 min	>3	[218]
*S. aureus*	Wood	Biosafety drying for 20 min	62.3 mg/L chlorine for 1 min	5.54	[250]
	Polypropylene			6	
Human norovirus	Stainless steel	Biosafety drying for 40 min	3% for 5 min	<2	[251]
Feline calicivirus	Stainless steel	Biosafety drying for 30 min	5.25% for 10 min	1.1	[238]
Feline calicivirus	Stainless steel	Drying for 1 h	12% (5000 ppm) for 5 min	5.20	[252]
Feline calicivirus	Polystyrene	Drying for 30 to 60 min	5.7% (100 ppm available chlorine) for 1 min	<2.27	[253]
Hepatitis A virus	Stainless steel	Biosafety drying for 1 h, in the presence of 5% soil	PAA 500 ppm for 10 min,	3.76	[231]
Murine norovirus	Stainless steel	Drying for 30 min	500 ppm for 5 min	<4	[54]
Murine norovirus	Stainless steel	Biosafety drying for 60 to 90 min	1350 ppm for 5 min	5.5	[254]
*Alicyclobacillus* spp. spores	Stainless steel	-	2000 ppm for 30 min	1.0	[249]

**Table 11:** Overview of disinfection methods used in the dry food industry.

Disinfection Method	Advantages	Disadvantages	Cost	Reference
Dry disinfection methods				
Brushing and scraping	Easily available	Laborious, not appropriate for all low-*a_w_* foods, small spectrum	Low	[104]
Isopropyl alcohol-quaternary ammonium-based disinfectants	Less concentration needed compared to QUAT, easily available	Harmful byproduct residue, potential for microbial resistance in certain strains	Comparable	[109,110]
Ethylene oxide gas fumigation	Higher coverage area, more penetrative depth	Shadowing effect, vapor or fog may not travel a greater distance, carcinogenic properties	Comparable/high	[114,115,116]
Methyl bromide gas fumigation	Broad antimicrobial spectrum, high efficiency	Contributes to ozone layer’s depletion, potent carcinogen	Low	[114,116]
Propylene oxide gas fumigation	Non-hazardous byproducts	Used with CO_2_ combination	Comparable	[118,119,121]
Chlorine dioxide gas fumigation	Greater penetration depth, fast action, on-site production, broad pH range	Unstable at higher concentrations, difficult to handle and transport	High	[133,134,143]
Ozone gas fumigation	Less environmental effect, broad antimicrobial spectrum, good coverage area	Unstable, harmful in gaseous state, poses greater health risk when exposed for longer time	Low/Comparable	[151,153,160,163]
Superheated steam	Non-polluting, fairly good antimicrobial spectrum, no toxic byproducts	Pre-cleaning required, efficiency depends on surface characteristics	High	[85,171]
UV-light disinfection	Non-thermal, less energy required, no odor	Shadowing effect, low penetrability	Low	[180,181,183]
Cold plasma	Non thermal, broad antimicrobial spectrum	Higher energy required for production, skilled labor required	Comparable/High	[57,185,193,196]
**Wet disinfection methods**				
Quaternary ammonium compounds	Convenient, broader pH range, non-corrosive	Can promote antibiotic resistance, formation of bacteriostatic film, incompatible with detergent	Low	[212,217,219]
Peracetic acid	Broad antimicrobial spectrum, environmentally friendly	Thermodynamically unstable, health concern at higher concentration, effectiveness varies with microorganism	Low	[38,223,224]
Hydrogen peroxide	Fast-acting, broad antimicrobial spectrum, no toxic byproducts, greater dispersion	Highly corrosive, highly sensitive to the presence of heavy metals	Low	[102,232,237]
Sodium hypochlorite	Convenient, broad antimicrobial spectrum	Requirement for handling precautions	Low	[155,243]
Electrolyzed water	Broad antimicrobial spectrum, lesser environmental impacts, no effect on sensory properties	Equipment corrosion, skin irritation, high initial cost, additional cleaning requirement	Comparable	[259,265]
Plasma activated water	Broad range of application, no hazardous byproducts, easy to use	Shorter lifespan of reactive species, cannot store for a longer time	Comparable/high	[269,270,272,273]

## Data Availability

No new data were required for this review manuscript.
